# A Comprehensive Account on Recent Progress in Pharmacological Activities of Benzimidazole Derivatives

**DOI:** 10.3389/fphar.2021.762807

**Published:** 2021-11-03

**Authors:** Shejuti Rahman Brishty, Md. Jamal Hossain, Mayeen Uddin Khandaker, Mohammad Rashed Iqbal Faruque, Hamid Osman, S. M. Abdur Rahman

**Affiliations:** ^1^ Department of Clinical Pharmacy and Pharmacology, Faculty of Pharmacy, University of Dhaka, Dhaka, Bangladesh; ^2^ Department of Pharmacy, State University of Bangladesh, Dhaka, Bangladesh; ^3^ Centre for Applied Physics and Radiation Technologies, School of Engineering and Technology, Sunway University, Bandar Sunway, Malaysia; ^4^ Space Science Centre, Universiti Kebangsaan Malaysia, Bangi, Malaysia; ^5^ Department of Radiological Sciences, College of Applied Medical Sciences, Taif University, Taif, Saudi Arabia

**Keywords:** nitrogenous heterocyclic compounds, diverse pharmacological activities, structure-activity relationship (SAR), anti-infectious, anti-proliferative, cardiovascular agents

## Abstract

Nowadays, nitrogenous heterocyclic molecules have attracted a great deal of interest among medicinal chemists. Among these potential heterocyclic drugs, benzimidazole scaffolds are considerably prevalent. Due to their isostructural pharmacophore of naturally occurring active biomolecules, benzimidazole derivatives have significant importance as chemotherapeutic agents in diverse clinical conditions. Researchers have synthesized plenty of benzimidazole derivatives in the last decades, amidst a large share of these compounds exerted excellent bioactivity against many ailments with outstanding bioavailability, safety, and stability profiles. In this comprehensive review, we have summarized the bioactivity of the benzimidazole derivatives reported in recent literature (2012–2021) with their available structure-activity relationship. Compounds bearing benzimidazole nucleus possess broad-spectrum pharmacological properties ranging from common antibacterial effects to the world’s most virulent diseases. Several promising therapeutic candidates are undergoing human trials, and some of these are going to be approved for clinical use. However, notable challenges, such as drug resistance, costly and tedious synthetic methods, little structural information of receptors, lack of advanced software, and so on, are still viable to be overcome for further research.

## Introduction

Benzimidazole, alternatively known as 1*H*-benzimidazole and 1,3-benzodiazole, consists of benzene ring fused with a five-membered imidazole ring, and is an important heterocyclic pharmacophore. Benzimidazole is regarded as a “privileged structure” in heterocyclic chemistry due to its association with a wide range of biological activities (Barot et al., 2013; Alaqeel, 2017).

**FIGURE 1:**
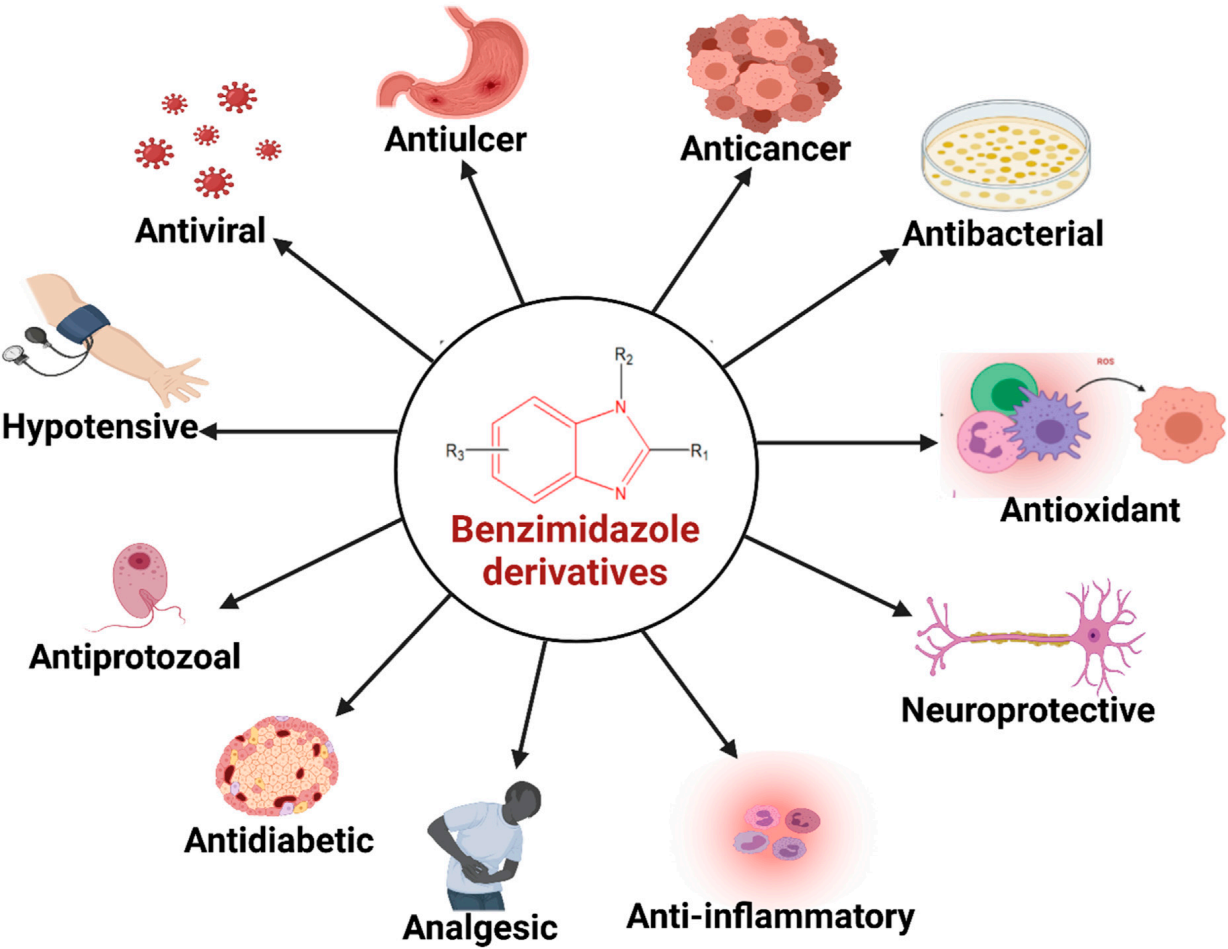
Diverse biological activities of benzimidazole derivatives.

**FIGURE 2:**
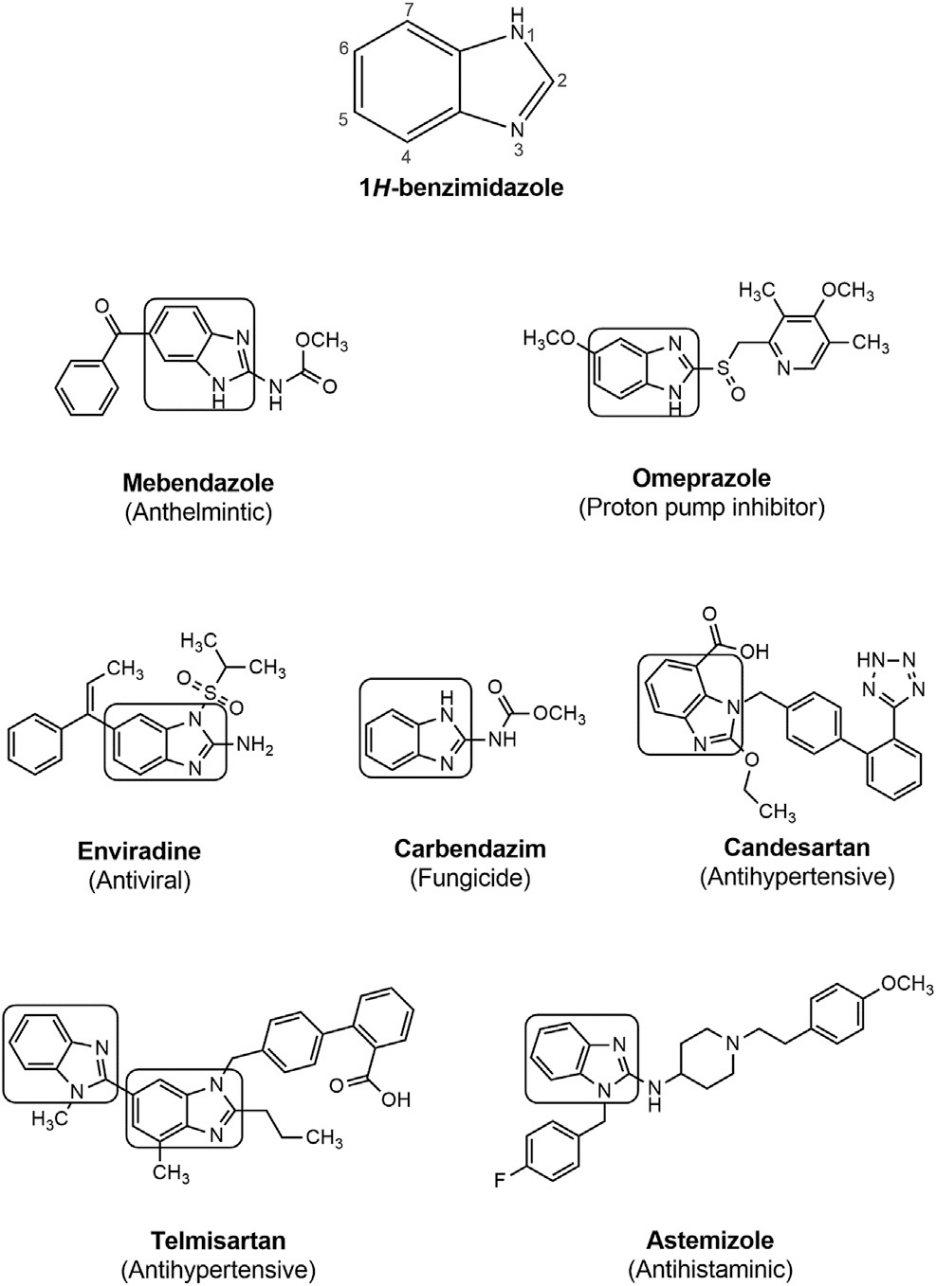
1*H*-benzimidazole and some benzimidazole containing drugs.

Back in 1940s, benzimidazole was speculated to act similarly as purines to provide biological responses and the first investigation on biological activity of benzimidazole nucleus was reported in 1944 (Woolley, 1944). Interest among the researchers about the synthetic procedure of benzimidazole and its derivatives escalated when Brink et al. (Brink and Folkers, 1949; Emerson et al., 1950) found that 5,6-dimethylbenzimidaozle was a degradation product of vitamin B_12_ and some of its derivatives also possessed vitamin B_12_ like activity. These early reports led researchers to the exploration of benzimidazole nucleus for numerous activities. Through the course of many years of research, benzimidazole has emerged as an important heterocyclic system because of its existence in diverse biologically active compounds, such as antiparasitics, antimicrobials, antivirals, antifungals, anticonvulsants, antihypertensives, antihistaminics, analgesics, anti-inflammatory agents, anticancers, anticoagulants and proton pump inhibitors (Figure 1) (Fei and Zhou, 2013; Wang et al., 2015). As a result of changing substituents around the core structure, many drugs of a wide variety of therapeutic lines have been developed such as albendazole, mebendazole, thiabendazole as antihelmintics; enviradine as antiviral; carbendazim as fungicidal; omeprazole, lansoprazole, pantoprazole as proton pump inhibitors; candesartan cilexitil and telmisartan as antihypertensives, and astemizole as antihistaminic agent (Figure 2) (Bansal and Silakari, 2012; Alaqeel, 2017). The high therapeutic potential of benzimidazole related drugs has inspired the medicinal chemists to carry out the synthesis of several novel chemotherapeutic agents containing benzimidazole moiety (Morais et al., 2017).

Numerous researches have been accomplished in the past couple of years which produced very intriguing results concerning the chemistry, structure-activity relationship and biological activities of different benzimidazole based compounds. The diverse biological activities displayed by compounds bearing benzimidazole moiety have prompted researchers all around the globe to design and synthesize various benzimidazole analogues. A number of recently published patents on the benzimidazole moiety are listed in Table 1. Several review articles have been published emphasizing on the contribution of benzimidazole nucleus in particular biological activity, e.g. anticancer, analgesic, anti-inflammatory, antimicrobial, antiviral, antitubercular, antiulcer, antihypertensive and antidiabetic property (Bansal and Silakari, 2012; Barot et al., 2013; Keri et al., 2015; Wang et al., 2015; Akhtar et al., 2017). To the best of our knowledge, there is no review article available in the literature which has focused on the most updated information of the diverse biological and therapeutic applications of benzimidazole derivatives. The present review gives a comprehensive account of all biological aspects of benzimidazole derivatives and has included information from the recent studies reported up to 2021. Apart from literature study, this review also provides a thoughtful insight into the latest ongoing research on benzimidazole derivatives in a variety of therapeutic fields.

**TABLE 1:** Recently published patents of benzimidazole derivatives.

Sl. No	Patent No	Country	Patent title	Publication date	Current status	Inventors	Brief description
1	ES2807191T3 Berrebi-Bertrand et al. (2021)	Spain	Benzimidazole derivatives as dual ligands of the histamine H1 receptor and the histamine H4 receptor	Feb 22, 2021	Active	Isabelle Berrebi-Bertrand, Xavier Billot, Thierry Calmels, et al	The compounds are supposed to have pharmaceutically acceptable salt, tautomers, hydrates, and solvation properties
2	AU2017382436A1 Crew et al. (2021)	Australia	Compounds and methods for the targeted degradation of Rapidly Accelerated Fibrosarcoma polypeptides	Jan 28, 2021	Active	Andrew P. Crew, Craig M. Crews, Hanqing Dong et al	The compound showed a wide range of biological activities by inhibition/degradation of target protein
3	US10835488B2 Pevzner and Moses-Heller (2020)	United States	Stable orally disintegrating pharmaceutical compositions	Nov 17, 2020	Active	Victor Pevzner, Sheera Moses-Heller	The composition has proton pump inhibition property
4	US10787420B2 Liu et al. (2020)	United States	Benzimidazole compound and preparation method thereof	Sep 29, 2020	Active	Xuejing Liu, Ying Han, Liang Yang	Several benzimidazoles have been synthesized through SN2 and cyclization reactions by utilizing no toxic reagent and/or any metal catalyst
5	US20190322671A1 Bourque and Skerlj (2019)	United States	Cxcr4 inhibitors and uses thereof	Oct 24, 2019	Pending	Elyse Marie Josee Bourque, Renato Skerlj	Data supported that the inventions have been regarded as CXCR4 blockers and healers of many diseases induced by the receptors
6	CA3079081A1 Bartberger et al. (2019)	Canada	Benzimidazole derivatives and their uses	April 25, 2019	Pending	Michael D. Bartberger, Nagasree Chakka, Hua Gao, et al	The inventions have been considered as Transient Receptor Potential Channel 6 (TRPC6) protein inhibitors
7	AU2020104192A4 Adak et al. (2018)	Australia	Process of synthesis of benzimidazole derivatives against M.tb	Dec 20, 2018	Active	Vishal Sudam Adak, Pravin Baburao Awate, Vishwas Chandrakant Bhagat, et al	The present invention have been disclosed as inhibitors of M.tb (H37Rv strain/ATCC No- 27294)
8	WO2018057810A1 Chandrasekhar et al. (2018)	France	Benzimidazole derivatives and their use as phosphatidylinositol 3-kinase inhibitors	Mar 29, 2018	NA	Jayaraman Chandrasekhar, Stephane Perreault, Leena Patel	The present compounds are acceptable salts, isomers, or a mixture thereof, which showed efficacy against various conditions of inflammation and cancer
9	US8372987B2 Kolaczkowski (2013)	United States	2-{(R)-2-methylpyrrolidin-2-yl)-1H-benzimidazole-4-carboxamide crystalline form 1	Sep 13, 2013	Active	Lawrence, Kolaczkowski	The present invention has notable application in facilitating DNA repair and controlling RNA transcription
10	US20150361032A1 Pajouhesh et al. (2015)	United States	Benzimidazole inhibitors of the sodium channel	Dec 17, 2015	Active	Hassan Pajouhesh Richard Holland, Lingyun Zhang, et al	The current patent represents benzimidazole derivatives that showed inhibition of voltage gated sodium channel, which might be promising in the treatment of various diseases and conditions
11	US 20150336967A1 Czardybon et al. (2015)	United States	Novel Benzimidazole Derivatives as Kinase Inhibitors	Nov 26, 2015	Active	Wojciech, Czardybon,Kraków Brzózka, Michal¸ Galezowski, et al	The patent describes benzimidazole derivatives as serine/threonine and tyrosine kinase-inhibitors with useful application in the treatment of solid tumors, lymphomas, leukaemia, and autoimmune disorders
12	US 20150322065A1 Chappie et al. (2015)	United States	Azabenzimidazole Compounds	Nov 12, 2015	Active	Thomas Allen Chappie, Patrick Robert Verhoest, Nandini, Chaturbhai Patel, Matthew Merrill Hayward	The present invention depicts the role of azabenzimidazole derivatives in the treatment of metabolic, central nervous system (CNS), autoimmune and inflammatory disorders
13	US 20150307479A1 Kuduk et al. (2015)	United States	Cyclobutyl benzimidazoles as pde 10 inhibitors	Oct 29, 2015	Active	Scott D. Kuduk, Casey C. McComas, Thomas S. Reger	The patent describes the usefulness of cyclobutyl benzimidazole derivatives in treating CNS disorders related to phosphodiesterase 10 (PDE10)
14	US 20150265625A1 Brown and Matthews (2015)	United States	(alpha-substituted aralkylamino and heteroarylalkylamino) pyrimidinyl and 1,3,5-triazinyl benzimidazoles, pharmaceutical compositions thereof, and their use in treating proliferative diseases	Sep 24, 2015	Active	S. David Brown, David J. Matthews	The current invention has useful application as drugs or agents in the treatment of proliferative diseases
15	US 20150218149A1 Apgar et al. (2015)	United States	Novel benzimidazole tetrahydrofuran derivatives	Aug 06, 2015	Active	James M. Apgar, Tesfaye Biftu, Ping Chen, Danqing Feng, Jacqueline D. Hicks, et al	The present invention suggests that novel benzimidazole tetrahydrofuran derivatives are effective against diseases mediated by the AMPK-activated protein kinase
16	US 20150209259A1 Schade et al. (2015)	United States	Octocrylene-free sunscreen composition with low stickiness	July 30, 2015	Active	Tatjana Schade, Kerstin Skubsch, Sina Brinkmann, et al	The present invention suggests an octocrylene-free cosmetic sunscreen composition which has low stickiness
17	US 20150203455A1 Menet et al. (2015)	United States	Novel compounds and pharmaceutical compositions thereof for the treatment of inflammatory disorders	July 23, 2015	Active	Christel Jeanne, Marie Menet, Oscar Mammoliti, Javier Blanc, et al	The patent describes novel benzimidazole derivatives in treating and preventing a number of inflammatory, autoimmune and proliferative disorders
18	US 20150175608A1 Tahri et al. (2015)	United States	Novel 4-substituted 1,3-dihydro-2h-benzimidazol-2-one derivatives substituted with benzimidazoles as respiratory syncytial virus antiviral agents	June 25, 2015	NA	Abdellah Tahri, Tim Hugo Maria Jonckers, Pierre Jean-marie Bernard, Raboisson, et al	The compounds have useful application as antiviral agents against respiratory syncytial virus (RSV)
19	US 20150175600A1 Atkinson et al. (2015)	United States	2-(azaindol-2-yl)benzimidazoles as pad4 inhibitors	June 25, 2015	Active	Stephen John, Atkinson, Michael David, Barker, Matthew, Campbell, et al	The patent describes the derivatives as PAD4 inhibitors with application against cystic fibrosis, rheumatoid arthritis, systemic lupus erythematosus, cancer, ulcerative colitis, asthma
20	US 20150158878A1 Leban and Zaja (2015)	United States	Bifluorodioxalane-amino-benzimidazole kinase inhibitors for the treatment of cancer, autoimmune inflammation and cns disorders	Jun 11, 2015	Active	Johann Leban, Mirko Zaja	The compounds are kinase inhibitors with notable role in the treatment of autoimmune inflammation, CNS disorders and cancer

## Biological Activities

The wide variety of benzimidazole derivatives synthesized during the last few years and their diverse biological applications are discussed in the following sections.

### Antimicrobial Activity

#### Antimicrobial and Antifungal Activity

**FIGURE 3:**
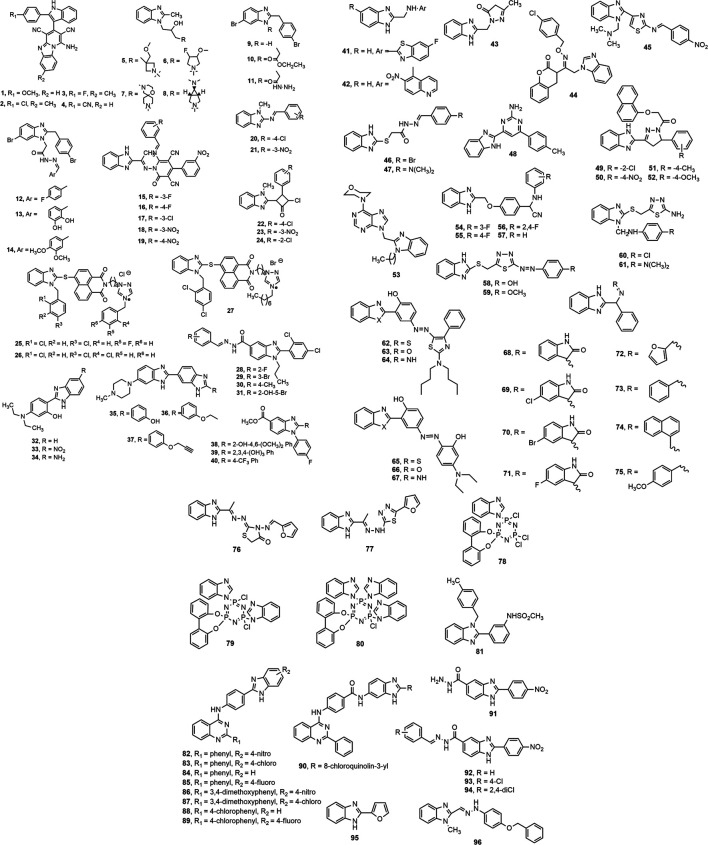
Benzimidazole derivatives with antimicrobial activity.

The antimicrobial potential of benzimidazole moiety has been explored notably since late 1990s and early 2000s (Özkay et al., 2011). Considering the huge dimension of research conducted on antimicrobial property of benzimidazole derivatives after 2012, the following section focuses on the up-to-date information on antibacterial and antifungal activities, while antiviral, antiulcer, antiprotozoal and antitubercular properties are discussed in separate sections. Different benzimidazole based compounds with antibacterial and antifungal activities are shown in Figure 3.

Kathrotiya and Patel (Kathrotiya and Patel, 2013) synthesized a series of indole-based pyrido [1,2-*a*] benzimidazole derivatives **(1–4)** and evaluated *in vitro* antimicrobial activity against some Gram-positive and Gram-negative bacteria and fungi using broth microdilution minimum inhibitory concentration (MIC) method. Compounds **1, 3** and **4** (MIC = 50, 62.5 and 12.5 μg/ml, respectively) displayed prominent antibacterial activity against *S. typhi* compared to standards ampicillin, chloramphenicol and ciprofloxacin (MIC = 100, 50 and 25 μg/ml, respectively). Compounds **2** and **3** exhibited notable antifungal activity against *C. albicans* (MIC = 250 μg/ml) in comparison with standard griseofulvin (MIC = 500 μg/ml). The derivatives with 4-methoxy **(1)** and cyano (**4**) group at 2-position of indole nucleus were found to possess excellent inhibitory activity against most of the tested organisms. Birajdar et al. (Birajdar et al., 2013) synthesized some amino alcohol derivatives of 2-methyl benzimidazole (**5–8)** by epoxide ring opening of 2-methyl benzimidazole with different substituted cyclic amines. Compounds **5–8** demonstrated moderate to good activity against Gram-positive (*S. aureus*) and Gram-negative (*E. coli*) pathogens in comparison with reference drugs ciprofloxacin and norfloxacin.

Several benzimidazole derivatives with imine functionality (**9–14)** were prepared by Kahveci et al. (Kahveci et al., 2014) using microwave irradiation as well as conventional method. Compounds **9–14** displayed notable antimicrobial property against the tested microorganisms. Desai et al. (Desai et al., 2014) synthesized a series of 2-pyridone based benzimidazole derivatives **(15–19)** and investigated *in vitro* antimicrobial potential against a number of bacterial and fungal strains using conventional broth dilution method. Compounds **15** and **18** (MIC = 12.5–25 µg/ml) showed better antibacterial activity, and **16** and **19** (MIC = 25–100 µg/ml) displayed comparable activity to standard chloramphenicol (MIC = 50 µg/ml). The presence of electron withdrawing groups, e.g. fluoro (**15, 16**) and nitro (**18, 19**) at the meta or para position might have contributed for their antimicrobial property. Compound **17** containing chloro group displayed the most remarkable antifungal activity, with MIC values in the range of 25–62.5 µg/ml against three fungal strains compared to standard ketoconazole (MIC = 50 µg/ml). A library of 1-methyl-*N*-[(substituted-phenylmethylidene)-1*H*-benzimidazol-2-amines **(20–24)** were synthesized and reported for notable antimicrobial activity against Gram-positive *S. aureus* (ATCC 6538), *B. pumilus* (ATCC 14884) and Gram-negative *E. coli* (NCTC 10418), *P. aeruginosa* (ATCC 25619) bacteria compared to reference drug ampicillin (Noolvi et al., 2014).

Luo et al. (Luo et al., 2015) synthesized a series of benzimidazole-based naphthalimide triazoles and triazolium compounds **(25–27)**. The derivatives were assessed for *in vitro* antibacterial activity against Gram-positive *S. aureus*, Methicillin-resistant *S. aureus*, *M. luteus* and *B. subtilis* and Gram-negative *B. proteus*, *E. coli*, *B. typhi*, and *P. aeruginosa* as well as for antifungal activity against *A. fumigatus*, *C. albicans*, *C. utilis*, *A. flavus*, and *S. cerevisiae*. The 2-chlorobenzyl triazolium compound **26** and octyl group-containing compound **27** showed the most potent antibacterial activity against *S. aureus* with an MIC of 2 μg/ml to standard norfloxacin (MIC = 2 μg/ml) and better than standard chloromycin (MIC = 7 μg/ml). Compound **26** and 3-fluorobenzyl moiety bearing compound **25** appeared to be the most prominent antifungal agents, with MIC value in the range of 2–19 μg/ml against the tested fungal strains. Vasantha et al. (Vasantha et al., 2015) synthesized a series of *N*-arylidene-2-(2,4-dichlorophenyl)-1-propyl-1*H*-benzo[d]imidazole-5-carbohydrazides derivatives among which compounds **28–31** displayed notable inhibitory effect against *A. niger* with MIC value of 3.12 μg/ml. Compound **28** demonstrated a MIC value of 3.12 μg/ml against most bacterial and fungal strains and appeared to be a potent antibacterial and antifungal agent.

A series of benzimidazole derivatives were synthesized and evaluated by Padalkar et al. (Padalkar et al., 2016) (**32–34**) and Chandrika et al. (Chandrika et al., 2016) (**35–37**), where the compounds **32–34** showed prominent antibacterial activity against *S. aureus* strain and compounds **35–37** were found to be the most potent antifungal agents against azole-resistant fungal strain *C. albicans* ATCC 64124 (strain B). Another study reported that among the total 22 synthesized novel 2-substituted fluorinated benzimidazoles, compounds **38–40** showed antimicrobial activity. In contrast, compound **40** containing a trifluoromethyl substituent showed the highest antifungal activity against the fungus *C. albicans* (Shintre et al., 2017). Similarly, El-Gohary and Shaaban (El-Gohary and Shaaban, 2017) synthesized a series of benzimidazole derivatives **(41–43),** where compounds **41** and **43** showed notable activity against *S. aureus*, and compound **42** was found to be the most effective against *B. cereus*. Compound **41** displayed the highest antifungal potential against *C. albicans*, whereas **43** exhibited prominent activity against *A. fumigatus*.

Singh et al. (Singh LR. et al., 2017) prepared a library of coumarin-benzimidazole hybrids and screened them for antimicrobial activity. Compound **44** was found to be the promising broad-spectrum antibacterial agent against *P. aeruginosa*, *S. aureus*, *B. subtilis* and *P. vulgaris*. A series of *N*-(substitutedbenzylidene)-4-(1-((dimethylamino)methyl)-1*H*-benzimidazol-2-yl)thiazol-2-amine derivatives were investigated for antimicrobial activity using agar streak dilution test. Compound **45** appeared to be the most potent among the series. Notably, the presence of an electron-withdrawing group might have contributed to the improved antimicrobial property of the compound (Prasad and Sundararajan, 2017).

Recently, Yadav et al. (Yadav et al., 2018) synthesized 2-substituted benzimidazole derivatives (**46–47**), where compound **46** emerged as the most potent antibacterial agent against both Gram-positive and Gram-negative bacteria compared to standard cefadroxil. All derivatives exhibited better antifungal activity than the standard fluconazole, and compound **47** showed maximum activity against *A. niger* (MIC = 0.018 mM). Liu et al. (Liu et al., 2018) designed a series of novel aminopyrimidinyl benzimidazoles as potential antimicrobial agents. Among them, compound **48** showed effective growth inhibition of MRSA, *E. coli,* and fungus *A. flavus*, compared to standard drugs chloromycin, norfloxacin, and fluconazole.

Another study reported the evaluation of a series of 1-(3-(1*H*-benzoimidazol-2-yl)-5-aryl-4-5dihydro-1*H*-pyrazol-1-yl)-2-(napthalene-1-yloxy) ethanones (**49–52**), where the electron-withdrawing groups (compound **49** containing chloro group at ortho position and **50** with a nitro group at the para position) were the most effective against bacterial strains. On the contrary, electron releasing groups at the para position (compounds **51** and **52** carrying methyl and methoxy group, respectively) contributed to the most promising antifungal activity against the tested organisms (Desai et al., 2018). Wang et al. (Wang et al., 2018) synthesized a library of purine benzimidazole hybrids and assessed them for antimicrobial potency. Compound **53** exhibited prominent activity against most tested bacterial and fungal strains and multidrug-resistant strain *S. aureus*, 16 times more potent than the standard norfloxacin (MIC = 4 µg/ml vs. 64 µg/ml). Amongst a series of α-aminonitrile based benzimidazole derivatives (**54–57**), the compounds **55** and **56** were found to be the most potent antibacterial agents having MIC values ranging between 3.9 and 7.8 μg/ml against different bacterial species. All the compounds exerted illustrious antifungal activity against *C. albicans* (MIC = 3.9–7.8 µg/ml) compared to the reference drug fluconazole (MIC value <3.9 µg/ml) (Shaikh et al., 2018).

Similarly, some recent publications also reported several potential antibacterial benzimidazole derivatives like compounds **58–61** containing the 1,3,4-thiadiazole ring and azo moiety showed excellent activity against *S. aureus, B. subtilis*, *E. coli*, and *p. aeruginosa* compared to amoxicillin and ciprofloxacin (Mahmoud et al., 2020). Also, the azo linked compounds **62–67** and novel Schiff bases of 2-(1-amino benzyl)-benzimidazole compounds **68–75** indicated moderate to high *in vitro* inhibition of both gram-positive (*S. aureus*) and gram-negative (*E. coli*) bacteria (Mishra et al., 2019; Singhal et al., 2019). Abdel-Motaal et al*.* (Abdel-Motaal et al., 2020) synthesized some substituted benzimidazole-2yl derivatives. Compounds **76** and **77** containing thiadiazole and thiazolone moieties, respectively, displayed antibacterial potency against *S. aureus, E. coli*, and *B. pumilus* comparable to standard gentamicin. Among the compounds **78–80**, the compound **80** highly inhibited the *B. subtilis* and *S. aureus* bacterial growth compared to the reference drug chloramphenicol (zone of inhibition, mm: 23 and 14 vs. 32 and 30) (İbişoğlu et al., 2020). Besides, co-treatment of compound **81** with colistin exhibited a promising synergistic effect against wild strains *E. coli*, *K. pneumoniae*, *A. baumannii*, and *P. aeruginosa* with MIC range = 8–16 μg/ml (Dokla et al., 2020). Malasala et al. (Malasala et al., 2021) reported nine more potent antibacterial agents **82–90** with MIC range 4–64 μg/ml against several resistant organisms, including methicillin and vancomycin-resistant *S. aureus*. The derivatives with 4-nitro, 4-chloro, 4-fluoro, 4-bromo, and unsubstituted exerted good to moderate inhibitory actions against *S. aureus and M. tuberculosis* H37Rv. Similarly, the analogues with phenyl, 3,4-dimethoxy, and 4-chloro exhibited moderate to good inhibitory property *S. aureus and M. tuberculosis* H37Rv (Malasala et al., 2021). Furthermore, compounds **91–94** inhibited *C. albicans* and *C. neoformans var. grubii* fungal growth with MIC values 4–16 μg/ml, and likewise, compounds **95** and **96** also exerted remarkable antifungal activity (Dhanamjayulu et al., 2019; Amine Khodja et al., 2020; Morcoss et al., 2020).

#### Antiviral Activity

**FIGURE 4:**
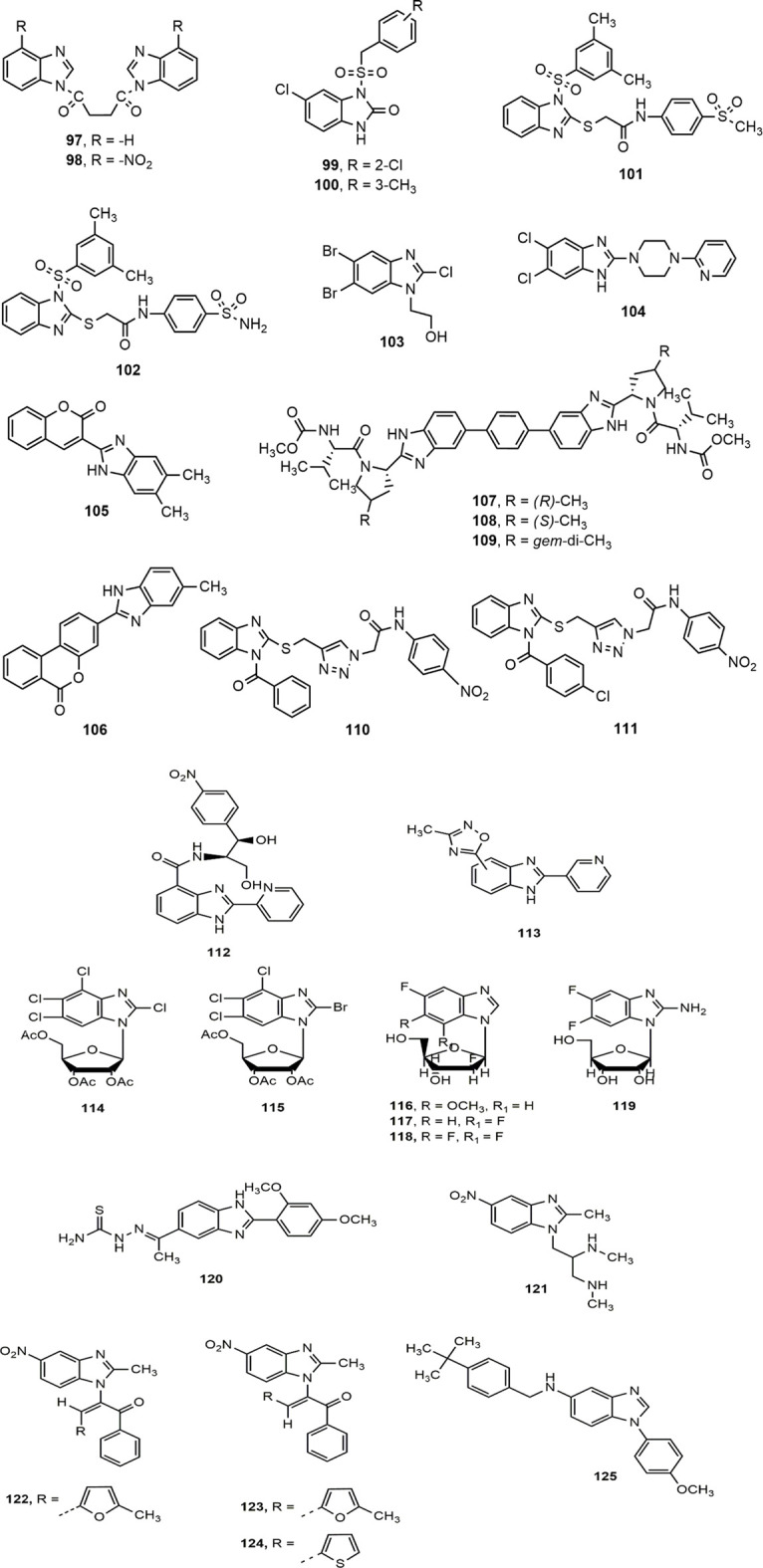
Benzimidazole derivatives with antiviral activity.

The antiviral properties of benzimidazole derivatives have been tested against different viral strains; human immunodeficiency virus (HIV), hepatitis B and C virus (HBV and HCV), enteroviruses, respiratory syncytial virus (RSV), human cytomegalovirus (HCMV), bovine viral diarrhea virus (BVDV) and herpes simplex virus-1 (HSV-1) are some to mention (Abu-Bakr et al., 2012). This section focuses on the recent studies involving varied antiviral properties of different benzimidazole derivatives, and their structures are shown in Figure 4.

##### Benzimidazole Against HIV

A number of substituted benzimidazole derivatives were synthesized as reverse transcriptase inhibitors (RTIs) against HIV-1 replication, among them compounds **97–98** showed notable antiviral activity against laboratory-adapted strains HIV-1_IIIB_ and HIV-1_Ada5_ (EC_50_ = 15.4–40 µM) and primary isolates of HIV-1_UG07O_ and HIV-1_VB59_ strains (EC_50_ = 5.28–31.86 µM) (Singh et al., 2015). Besides, Ferro et al. (Ferro et al., 2017) synthesized two series of N_1_-aryl-benzimidazol-2-one derivatives as non-nucleoside reverse transcriptase inhibitors (NNRTIs) against HIV-1, where the compounds **99–100** were more potent than the standard drug nevirapine (IC_50_: 1.3 and 0.79 vs. 1.55 µM). The sulfone derivatives **101–102**, synthesized by the same research group were also found to be potent HIV-1 NNRTIs with IC_50_ values of 47 and 50 nM, respectively. The substitution at C-4 position of the arylacetamide portion of the compounds might have contributed for their notable activity against HIV-1_IIIB_ strain in cell-based assays (Monforte et al., 2018). Finally, Srivastava et al. (Srivastava et al., 2020) has recently reported a promising anti-HIV benzimidazole derivative **103** with a low IC_50_ value of 0.386 × 10^–5^ μM.

##### Benzimidazole Against Hepatitis B and C Viruses (HBV and HCV)

The hepatitis B surface antigen (HBsAg), an HBV surface protein is an important mediator of HBV life cycle (Wang et al., 2016). Xu et al. (Xu et al., 2014) carried out a high-throughput screening (HTS), and concluded that the compound **104** inhibited the secretion of HBsAg and HBV virions indicated by EC_50_ values of 1.5 and 0.6 μM, respectively, along with half cytotoxicity concentration (CC_50_) value of 24.5 μM.

Moreover, Tsay et al. (Tsay et al., 2013) synthesized a library of hinged benzimidazole-coumarin hybrids and reported the potentiality of compounds **105–106** with EC_50_ values 3.0 and 5.5 nM, respectively, against hepatitis C virus (HCV), a prime cause of liver cirrhosis and hepatocellular carcinoma. Besides, Henderson et al. (Henderson et al., 2015) synthesized a series of 4-substituted pyrrolidine containing bis-benzimidazole analogues (**107–109**) and evaluated for their HCV non-structural 5A (NS5A) inhibitory effect with balanced Genotype 1a (G1a) and Genotype 1b (G1b) potency. Compounds **107** (G1a EC_50_ = 0.028 nM, G1b EC_50_ = 0.007 nM), **108** (G1a EC_50_ = 0.026 nM, G1b EC_50_ = 0.037 nM) and **109** (G1a EC_50_ = 0.03 nM, G1b EC_50_ = 0.011 nM) appeared to be most active compounds from the series. Substitution of methyl group (**107–108**) and geminal dimethyl group (**109**) at 4-position of pyrrolidine nucleus was likely to contribute for G1a and G1b potency of the compounds. In another study, a set of 2-thiobenzimidazole analogues (**110–111**) containing triazole moiety were reported as promising HCV inhibitor, where the substituent at position-2 of benzimidazole nucleus played the crucial role to enhance the antiviral potency (Youssif et al., 2016).

##### Benzimidazole Against Enteroviruses, Cytomegalovirus and Herpes Simplex Virus (HSV)

Two distinct series of benzimidazole derivatives were synthesized, where the compounds **112–113** indicated potent enterovirus (Coxsackie) inhibition with the IC_50_ values of 1.76 and 1.08 μg/ml, respectively (Xue et al., 2011; Wubulikasimu et al., 2013). The benzimidazole D-ribonucleosides derivatives **114–115** exerted activity in rat cytomegalovirus infected cells, and prevented cleavage of concatemeric viral DNA and nuclear egress of mature viral capsids (Dittmer et al., 2017). In a pair consecutive studies (Kharitonova et al., 2016, 2017), Kharitonova et al. reported that several 2′-deoxy-2′-fluoro-β-D-arabinofuranosyl benzimidazole derivatives **(116–118)** and 2-amino-5,6-difluorobenzimidazole nucleosides **(119)** inhibited Herpes Simplex Virus-induced cytopathic effect (CPE). Impressively, the IC_50_ value of compound **119** was 104 μM, four times lower than that of ribavirin and eight times lower than that of maribavir.

##### Benzimidazole Against Bovine Viral Diarrhea Virus (BVDV), Rotavirus and Arenaviruses

Among a library of 5-acetyl-2-arylbenzimidazoles analogues, compound **120** appeared to be the most effective antiviral agent against Bovine Viral Diarrhea virus (BVDV, EC_50_ = 1.11 mM) due to the presence of 2,4-dimethoxy group in the phenyl moiety (Vitale et al., 2012). Shaker et al. (Shaker et al., 2015) synthesized a series of 5-nitro-1*H*-benzimidazole derivatives (**121–124**) bearing substitution of heterocyclic rings at position 1. Compounds **121** and **122** exhibited equal potency as standard doxorubicin against A-549, HCT-116, MCF-7 and human liver carcinoma HepG2 cell lines. Besides, compounds **122–124** showed great potential to be used as potent antiviral agents due to their inhibitory effect against rotavirus Wa strain. Finally, compound **125**, identified by Dai and co-workers, was found to be potent antiviral agent against Lassa virus envelope glycoprotein (LASV GP) pseudotypes with EC_50_ value of 1.1 nM (Dai et al., 2013).

### Anti-inflammatory and Analgesic Activity

**FIGURE 5:**
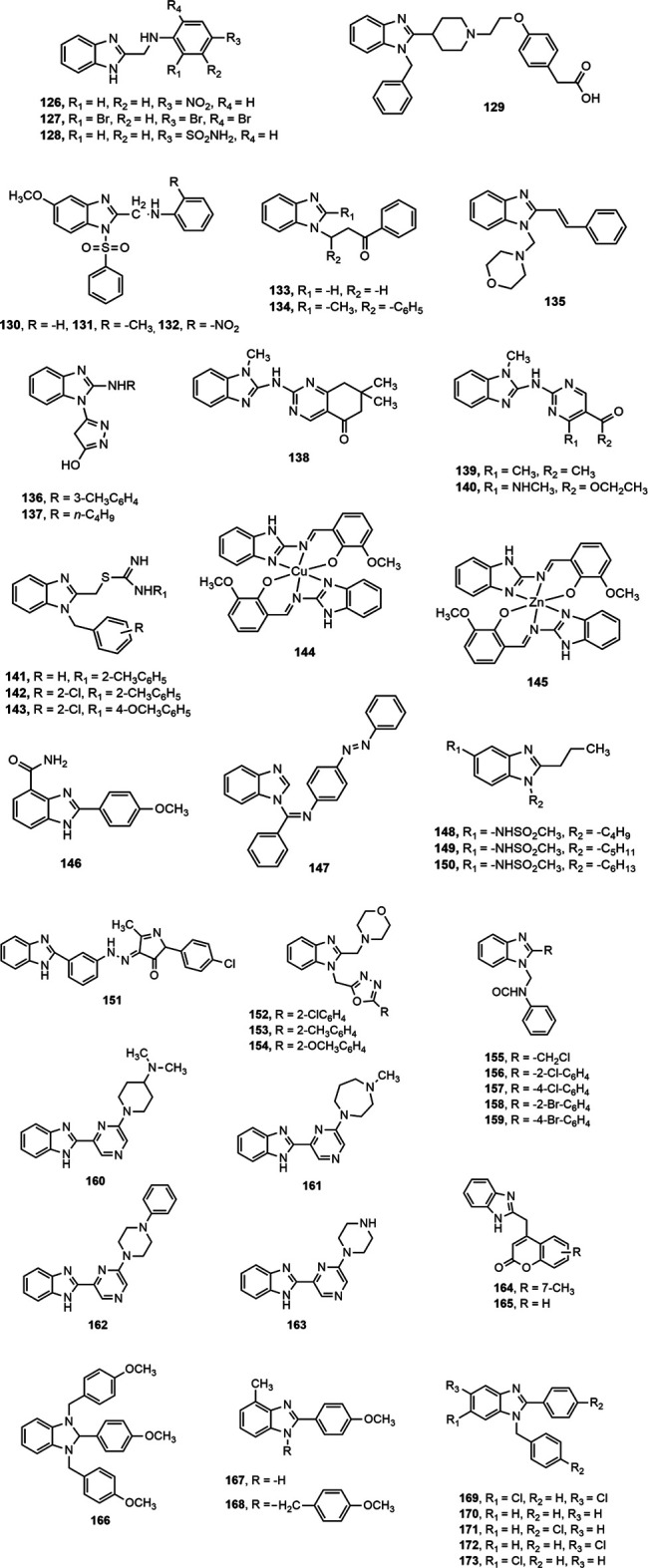
Benzimidazole derivatives with anti-inflammatory and analgesic activity.

Benzimidazole based compounds are of great importance as anti-inflammatory and analgesic agents because of their property to inhibit cyclooxygenases (COXs), enzymes involved in biosynthesis of important inflammatory mediators called prostaglandins (Akhtar et al., 2017). Apart from the cyclooxygenases (COX), the benzimidazole derivatives interact with transient receptor potential vanilloid-1, cannabinoid receptors, bradykinin receptors, specific cytokines, and 5-lipoxygenase (5-LOX) activating protein. Thus, the compounds derived from benzimidazole moiety show the anti-inflammatory property (Veerasamy et al., 2021). Different benzimidazole derivatives with analgesic and anti-inflammatory properties are shown in Figure 5.

Mariappan et al. (Mariappan et al., 2015) synthesized a set of 2-substituted benzimidazole derivatives and reported that compounds **126–128** were found to be the most promising agents among the series displaying significant (*p* < 0.01) analgesic and anti-inflammatory effect at a dose level of 100 mg/kg p.o. Li et al. (Li et al., 2015) assessed the synthesized two series of 2-(piperidin-4-yl)-1*H*-benzo[d]imidazole derivatives for anti-inflammatory activity and found that the compound **129** exhibited the most potent inhibitory activity on nitric oxide and TNF-α production (IC_50_ = 0.86 and 1.87 µM, respectively). Interestingly, the compound **129** also prevented 33.30 and 50.60% ear oedema in xylene-treated mice at doses of 4 and 12 mg/kg, respectively, compared to standard ibuprofen (26.77 and 39.34% inhibition at 4 and 12 mg/kg dose levels, respectively). Besides, compounds **130–132** showed the potentiality as gastroprotective lead compounds which can be developed into orally active analgesic and anti-inflammatory agents (Gaba and Mohan, 2015). Compounds **133–134,** synthesized by Kumar et al. displayed significant analgesic and anti-inflammatory properties, (Kumar et al., 2015) and compound **135** showed better inhibition of acetic acid induced writhing at 20 mg/kg dose than the standard diclofenac (78.12 vs. 75%) (Datar and Limaye, 2015).

Moneer et al. (Moneer et al., 2016) synthesized a series of 5-[2-(substituted amino)-1*H*-benzimidazol-1-yl]-4*H*-pyrazol-3-ol derivatives (**136–137**) and investigated for *in vitro* cyclooxygenase inhibitory effect using Cayman’s colorimetric COX (ovine) assay. Compounds **136–137** showed remarkable *in vitro* cyclooxygenase inhibition (IC_50_ on COX-1: 0.1664 and 0.2272 nM, respectively, and IC_50_ on COX-2: 0.0370 and 0.0469 nM, respectively) and significant (*p* < 0.05) reduction of edema volume compared to that of standard diclofenac at all time intervals. Prajapat and Talesara (Prajapat and Talesara, 2016) synthesized several alkoxyphthalimide based benzimidazole derivatives (**138–140**) and Siddiqui et al. (Siddiqui et al., 2016) prepared a series of 1-{(1-(2-substituted benzyl)-1*H*-benzo[d]imidazol-2-yl)methyl}-3-arylthioureas (**141–143**). All the compounds **(138–143)** exerted notable anti-inflammatory effect compared to standard diclofenac.

**FIGURE 6:**
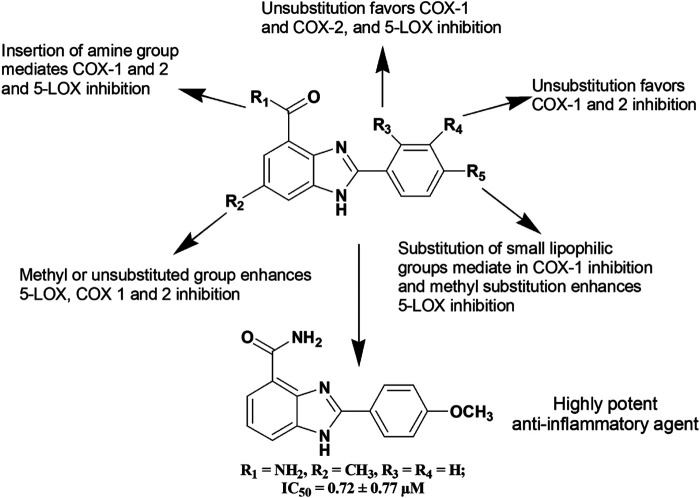
Structure activity relationship (SAR) of benzimidazole derivatives having anti-inflammatory activity. The figure represents the SAR studies accomplished by Bukhari et al. (2016).

A series of Cu(II) and Zn(II) complexes of a 2-[(1*H*-benzoimidazol-2-ylimino)-methyl]-6-methoxy-phenol Schiff base ligands (**144–145**) were synthesized from condensation of 2-aminobenzimidazole and *o*-vanillin (AlAjmi et al., 2016). The both compounds (**144–145)** at 100 mg/kg b.w. showed around 55% inhibition of inflammation compared to standard diclofenac (65.4% inhibition). Furthermore, Bukhari et al. (Bukhari et al., 2016) reported about anti-inflammatory activity of a series of benzimidazole derivatives where compound **146** was found to be a potent inhibitor of 5-LOX, COX, TNF-α, IL-6 and cytokines among the series. Figure 6 depicts a clear diagram for understanding the association of structural modifications with bioactivities of benzimidazole derivatives against inflammation.

Eswayah et al. (Eswayah et al., 2017) synthesized several *N*-substituted benzimidazole derivatives and concluded that compound **147** exhibited prominent analgesic activity indicated by decrease in number of writhing at 50 mg/kg dose compared to standard aspirin (17 vs. 12%). Besides, Sharma et al. (Sharma R. et al., 2017) stated that compounds **148–150** demonstrated notable reduction in edema ranging from 92.7 to 97.6% compared to the standard drugs rofecoxib and indomethacin (78.95 and 75%, respectively). Moreover, compound **151** displayed 72% analgesic activity and 67% protection of inflammation at 20 mg/kg dose in second hour in comparison with standard diclofenac (69% analgesic activity and 65% protection of inflammation) (Chikkula and Sundararajan, 2017). Similarly, Rathore et al. (Rathore et al., 2017) synthesized a series of 1-{(5-substituted-1,3,4-oxadiazol-2-yl)methyl}-2-(morpholinomethyl)-1*H*-benzimidazole derivatives (**152–154**) and reported that the compound **152** having chloro group at the ortho position of phenyl ring showed promising anti-inflammatory effect compared to standard indomethacin (74.17 ± 1.28% vs. 57.79 ± 1.71%). Besides, compounds **152–154** also produced remarkable COX-2 inhibition (IC_50_ range = 8–13.7 µM).

By applying Mannich reaction, Sethi et al. (Sethi et al., 2017) prepared a series of *N*-benzimidazol-1-yl methyl-benzamide derivatives (**155–159**), having electron-withdrawing groups chloro and bromo at ortho position of phenyl ring (**156–158**) and chloromethyl group at 2-position of benzimidazole nucleus (**155**), asserted significant analgesic and anti-inflammatory activities compared to vehicle control group (10% DMSO, *p* < 0.05). Moreover, Shankar et al. (Shankar et al., 2017) synthesized a series of 2-(6-alkyl-pyrazin-2-yl)-1*H*-benzo[d]imidazole derivatives (**160–163**), where the compound **162** showed maximum selectivity towards COX-2 enzyme among the series (% inhibition 78.68 ± 0.46 and selectivity ratio 3.71) and it might be due to the presence of *N*-phenyl piperzine moiety in the benzimidazole nucleus. Besides, compounds **160, 161** and **163** demonstrated notable activity against COX-2 enzyme (% inhibition 71.45 ± 0.65, 76.93 ± 0.84 and 58.27 ± 0.25, respectively).

Recently, Sethi et al. (Sethi et al., 2018) synthesized two series of benzimidazole based compounds from the coupling of coumarin and benzimidazole nuclei and narrated the anti-inflammatory activity of compounds **164–165** compared to the standard indomethacin (45 vs. 48%). Brishty et al. (Brishty et al., 2020) synthesized a group of substituted benzimidazole derivatives amongst which compounds **166, 167** and **168** exhibited remarkable analgesic activity by inhibition of acetic acid induced writhing of mice compared to standard diclofenac (88.24, 84.03 and 85.71%, respectively, vs. 90.76%; *p* < 0.001). In continuation of the research by the group, Saha et al. (Saha et al., 2020) prepared a number of disubstituted benzimidazole derivatives. Compounds **169, 170** and **171** displayed notable analgesic property at a dose of 25 mg/kg by 88.81, 69.40 and 64.93% writhing inhibition, respectively (*p* < 0.05) in comparison with standard aceclofenac (88.81%). Very recently, the group reported the pharmacological investigation of some of their previously synthesized benzimidazoles (Saha et al., 2021). Compounds **170, 172** and **173** exhibited promising central analgesic potential in radiant heat tail flick method compared to standard morphine (% of elongation 58.07, 51.59, and 76.65, respectively vs. 87.17). Besides, **171, 172** and **173** displayed notable reduction in paw edema (81.75, 79.09 and 86.69%, respectively) comparable to standard aceclofenac (87.83) (Saha et al., 2021).

### Antiulcer Activity

**FIGURE 7:**
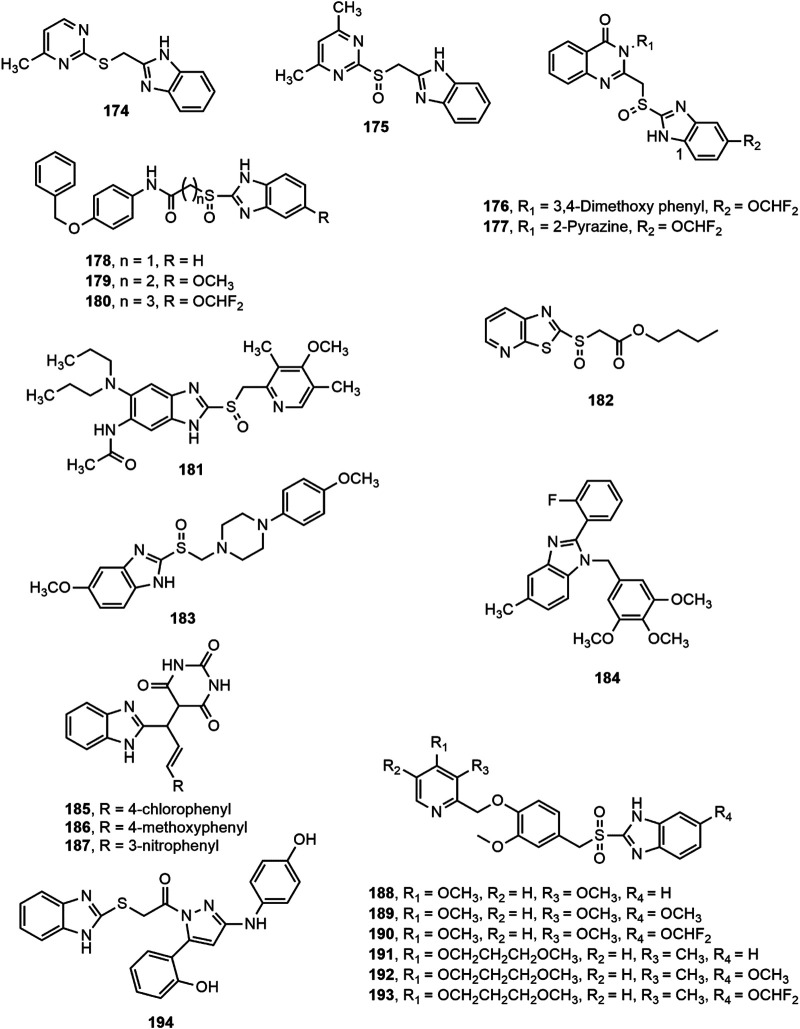
Benzimidazole derivatives with antiulcer activity.

Many benzimidazole derivatives are known to possess potent antiulcer activity and H^+^/K^+^-ATPase inhibitory properties. During recent times, several new synthetic benzimidazole-based compounds were developed which exhibited similar or better antiulcerogenic potentials compared to the established market preparations. The benzimidazole derivatives with antiulcer activity are shown in Figure 7.

A series of pyrimidylthiomethyl benzimidazoles (e.g. **174**) and pyrimidylsulfinylmethyl benzimidazole derivatives (e.g. **175)** were developed and screened for antiulcer activity. Compounds **174–175** significantly reduced gastric acid secretion, free acidity and gastric ulcers in the pylorus-ligated rats at 10 and 30 mg/kg doses, where the sulfinyl derivative (**175**) was found to be more effective than thio derivative (**174**) (Bariwal et al., 2008). In another study, compounds **176–177** exhibited most prominent antiulcer activity against pylorus ligation-induced, aspirin induced, and ethanol induced ulcer in rat model at a dose level of 10 and 20 mg/kg compared to omeprazole (Patil et al., 2010). Besides, Reddy et al. (Reddy et al., 2011) prepared a series of 2-substituted mercaptobenzimidazole derivatives and reported that compounds **178–180** produced notable antiulcer potentiality at a dose level of 10 mg/kg comparable to omeprazole. Furthermore, compound **181** prevented H^+^/K^+^-ATPase enzymatic activity with an IC_50_ value of 1.6 × 10^–5^ M and compound **182** displayed prominent effects on inhibition of gastric lesions and gastric acid secretion in a dose dependant manner (0.3–30 mg/kg) (Tanaka et al., 2011; Yan et al., 2011).

Moreover, some benzimidazole-piperazine conjugated analogues were assessed for their *in vivo* antiulcer property. The 4-methoxy phenyl piperazine substituted benzimidazole derivative **(183)** appeared to be the most effective agent (Patil et al., 2012). Chang et al. (Chang et al., 2012) developed a series of 3,4,5-trimethoxybenzylbenzimidazole derivatives among which compound **184** (2-fluorophenyl-5-methyl-1-(3,4,5-trimethoxybenzyl) benzimidazole) emerged as the most potent inhibitor of *Helicobacter pylori* growth and pathogenesis of host cells. The compound specifically inhibited *H. pylori* adhesion and invasion of gastric epithelial cells, as revealed by *in vitro H. pylori* infection model. Mathew et al. (Mathew et al., 2013) synthesized a series of substituted benzimidazole derivatives (**185–187**) and reported that derivatives **185–187** exerted remarkable protection of ulcer (69.58, 69.56 and 67.17%, respectively) at a dose of 50 mg/kg b.w compared to omeprazole (77.37%, 2 mg/kg b.w.). Amongst a series of substituted methoxybenzyl-sulfonyl-1*H*-benzo[d]imidazole derivatives, compounds **188–193** appeared to be the most potent H^+^/K^+^-ATPase inhibitors compared to omeprazole (Rajesh et al., 2017). Finally, some new benzimidazole-pyrazole hybrids were evaluated for *in vivo* anti ulcerogenic activity using ethanol-induced gastric ulcer model in Albino rats. Compound **194** was found to be the most potent among the series with 83.1% ulcer inhibition at a dose level of 500 μg/kg (Noor et al., 2017).

### Antioxidant Activity

**FIGURE 8:**
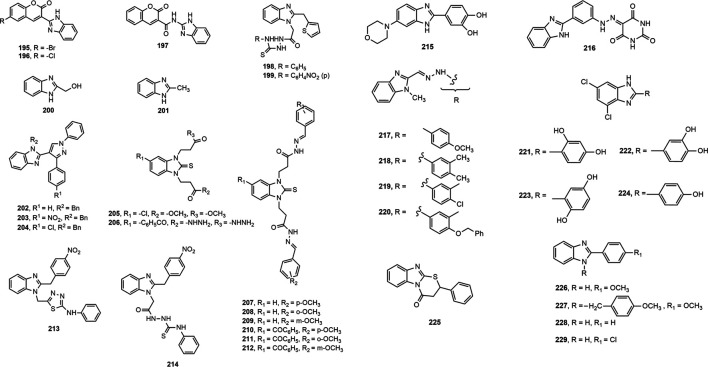
Benzimidazole derivatives with antioxidant activity.

Several benzimidazole derivatives have been explored through years for their capacity to act as antioxidants. Different benzimidazole derivatives with antioxidant activity are shown in Figure 8.

Two series of benzimidazole compounds made through coupling of coumarin derivatives with benzimidazole nucleus either directly or through amide linkage at 2-position were evaluated for antioxidant property (Arora et al., 2014). Compounds **195–197** demonstrated excellent antioxidant activity (IC_50_ values 19.7, 13.9 and 1.2 µmol/L, respectively) compared to standard butylated hydroxytoluene (BHT, IC_50_ = 23.4 µmol/L). Among the different benzimidazole derivatives with heterocyclic moieties developed by Mentese et al. (Menteşe et al., 2015), compound **198–199** with a thiophene ring exhibited remarkable antioxidant activity. Poddar et al. synthesized and evaluated the antioxidant property of substituted benzimidazoles by 1,1-diphenyl-2-picrylhydrazyl (DPPH) free radical scavenging method (Poddar et al., 2016). Compound **200** and **201** exhibited mild to moderate antioxidant potential (IC_50_ = 400.42 and 144.84 µg/ml, respectively) in comparison with standard BHT (IC_50_ = 51.56 μg/ml).

Among a library of *N*-substituted pyrazole-containing benzimidazoles, compounds **202**–**204** attributed prominent antioxidant activity in both DPPH and hydrogen peroxide assay supposed to the presence of benzyl substituent on imidazole nitrogen (Bellam et al., 2017). Besides, Anastassova et al. (Anastassova et al., 2018a) evaluated antioxidant property of compounds **205–206** using tert-butyl hydroperoxide (*tert*-BOOH) induced oxidative stress on rat hepatocytes, and reported that both compounds showed significant effect comparable to standard quercetin. Similarly, compounds **207–212** displayed notable cytoprotective effect on rat hepatocytes (Anastassova et al., 2018b).

Moreover, a library of 2-(4-nitrobenzyl)-1*H*-benzimidazole derivatives showed good antioxidant property where compounds **213** and **214** demonstrated prominent inhibitory effect against xanthine oxidase (IC_50_ = 12.30 ± 0.33 μg/ml) and urease (IC_50_ = 13.04 ± 0.89 μg/ml), respectively (Karaali et al., 2018). Furthermore, compound **215** had the most potency in the series of 2-(aryl)-6-morpholin-4-yl(or 4-methylpiperazin-1-yl)-1*H*-benzimidazoles (Özil et al., 2018).

**FIGURE 9:**
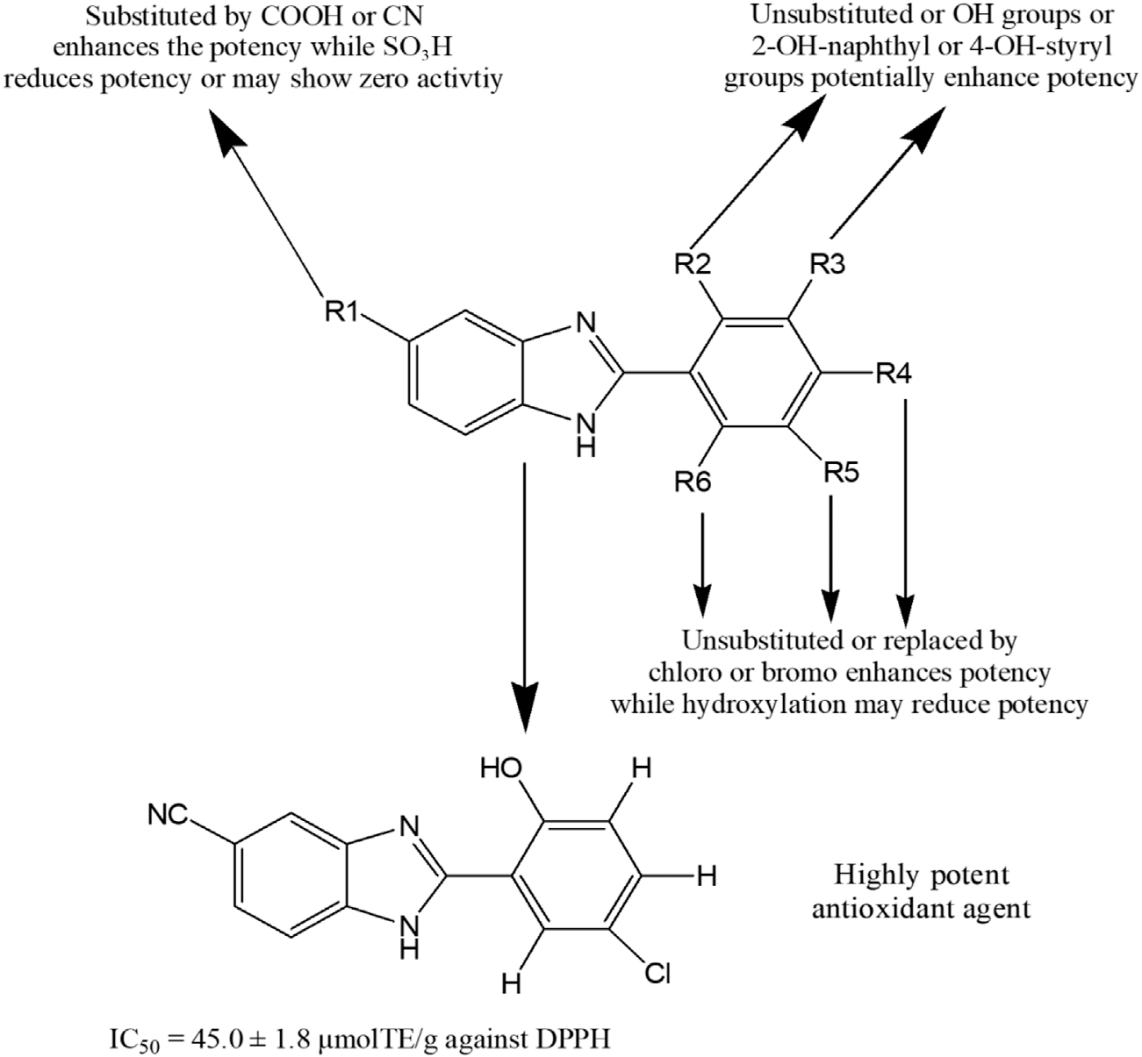
Structure activity relationship (SAR) of benzimidazole derivatives having antioxidant potentiality. The figure represents the SAR studies accomplished by Baldisserotto et al. (2020).

Recently, Baldisserotto et al. (Baldisserotto et al., 2020) synthesized total 39 arylbenzimidazole derivatives and reported their remarkable potency against various free radicals. Figure 9 represents a general structure for describing SAR of benzimidazoles possessing antioxidant activity. The addition of cyano or carboxyl group at 5-position (R_1_) of the benzimidazole nucleus was responsible for exhibiting medium to high potency against several free radicals. In contrast, the derivatives containing the 5-sulfonic acid group showed poor or no antioxidant properties. Unsubstituted 2-aromatic ring or OH, Cl, Br, 2-OH-napthyl or 4-OH-steryl substitutions enhanced activity. In another study, compound **216** showed 40–80% antioxidant potential at different concentrations (10–100 µM) (Abdelgawad et al., 2019). Amine Khodja et al. reported that compounds **217–220** exerted promising inhibition capacity against various free radicals compared to BHT (IC_50_ (mean ± SD, µM) for DPPH assay: 40.4 ± 0.9 to 60.4 ± 1.9 vs. 70.8 ± 6.6) (Amine Khodja et al., 2020). Taha et al. (Taha et al., 2020) synthesized 20 benzimidazole derivates and found four potent antioxidant compounds **221–224** with IC_50_ values (mean ± SEM: 22.42 ± 0.26 to 40.60 ± 0.80) comparable to standard propyl gallate (29.20 ± 1.25). Interestingly, compound **225** exhibited more inhibition (%) of DPPH-free radical than the standard antioxidant Trolox (73% ± 2.42 vs. 70% ± 0.35) (Ramos Rodríguez et al., 2020). Furthermore, compounds **166, 168, 226** and **227** displayed prominent antioxidant property with lower IC_50_ values than the standard BHT (8.834, 7.519, 0.038 and 0.959 μg/ml, respectively vs. 14.44 μg/ml) (Brishty et al., 2020)**.** Finally, compounds **228** and **229** demonstrated mild antioxidant potential in comparison with standard ascorbic acid (IC_50_ = 12.25 × 10^3^ and 87.326 × 10^3^ μg/ml, respectively vs. 2.19 μg/ml) (Saha et al., 2021).

### Anticancer Activity

**FIGURE 10:**
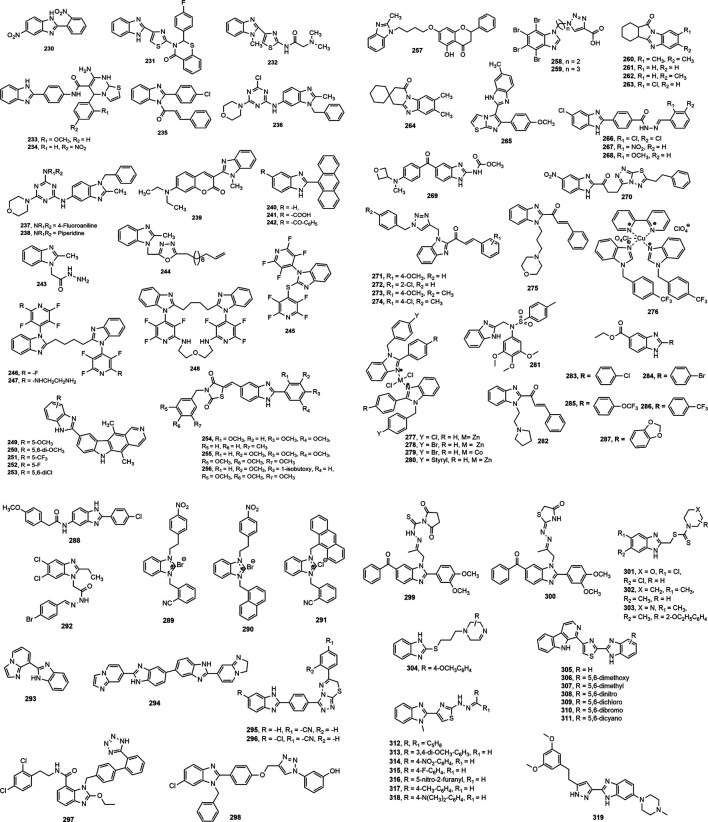
Benzimidazole derivatives with anticancer activity.

Among the anticancer drugs discovered in the recent years, different benzimidazole derivatives occupy an important place. The current review accounts the anticancer activity of benzimidazoles reported after 2013. The benzimidazole derivatives with anticancer activity are shown in Figure 10.

A series of substituted benzimidazole derivatives were evaluated for *in vitro* anticancer activity in human lung adenocarcinoma A549 cell line at normoxic and hypoxic conditions. Compound **230** was found to be the most cytotoxic agent with hypoxia/normoxia cytotoxic coefficient of 4.75, compared to standard tirapazamine (5.59) (Błaszczak-Świątkiewicz et al., 2014). The benzimidazole-thiazole derivatives **231–232** showed notable anticancer effect against human liver carcinoma cell line (HepG2: IC_50_ = 0.518 and 0.578 mM) and pheochromocytoma of the rat adrenal medulla cell line (PC12: IC_50_ = 0.309 and 0.298 mM) (Nofal et al., 2014). Compounds **233–234** with dual inhibition of Aurora A kinase and kinesin spindle protein were found to be the most prominent antitumor agents against various tested cell lines in comparison with standard drug CK0106023 (Abd El-All et al., 2015). Kalalbandi and Seetharamappa synthesized a series of 1-[(2*E*)-3-phenylprop-2-enoyl]-1*H*-benzimidazole derivatives among which compound **235** displayed notable antiproliferative activity against nine tumor subpanels, indicated by its selectivity ratios within the range of 0.79–1.53 and 0.47 to 1.69 at the GI_50_ (growth inhibitory 50%) and TGI level, respectively (Kalalbandi and Seetharamappa, 2015). Similarly, compounds **236–238** with GI_50_ values of 9.79, 2.58 and 3.81 µM, respectively exhibited broad spectrum antitumor activities, while the compound **236** was found to be the most potent DHFR inhibitor with IC_50_ value of 1.05 µM (Singla et al., 2015).

Among the derivatives identified by Liu et al. (Liu et al., 2015), compound **239** showed notable inhibition of PI3K-AKT-mTOR pathway having GI_50_ values in the range of 0.07–0.41 μmol/L against most of the tested cancer cell lines, signifying its potential to be used as anticancer agent. Sontakke and co-workers (Sontakke et al., 2015) synthesized 2-anthryl benzimidazole derivatives (**240–242**) bearing hydrogen, benzoyl and carboxyl substituents, respectively at 5^th^ position. Compounds **241** and **242** showed anticancer potency against MCF-7 cell lines (IC_50_: 16.18 and 19.21 µM, respectively) and HL-60 cell lines (IC_50_: 15.15 and 18.29 µM, respectively) followed by compound **240** (MCF-7 IC_50_ = 20.48 µM and HL-60 IC_50_ = 23.23 µM). In another study, compound **243** displayed most notable activity against HeLa cell lines (IC_50_ = 05.34 ± 1.2 µM) compared to standards doxorubicin and 5-flurouracil (IC_50_ = 03.56 ± 2.7 and 02.78 ± 2.6 µM, respectively). On the other hand, compound **244** showed marked activity against Hep3B cell line with an IC_50_ value of 11.10 ± 1.1 µM (Varshney et al., 2015).

Bhambra et al. (Bhambra et al., 2016) synthesized a library of fluoroaryl benzimidazole derivatives (**245–248**) among which compounds **246–248** demonstrated inhibition against K-562 and MCF-7 cell lines in micromolar range. Compounds **246** and **248** were reported as activators of caspases, which play important role in apoptosis of cancerous cells. Bramhananda Reddy et al. designed and synthesized a library of benzimidazole fused ellipticine derivatives (**249–253**) and delineated antiproliferative potential against human cancer cell lines Zr-75–1, HeLa, MCF-7 and A-549 with GI_50_ values of <0.1–34.6 µM, compared to standard etopoxide (GI_50_ = 0.2–3.08 µM) (Bramhananda Reddy et al., 2016). Sharma et al. (Sharma P. et al., 2017) designed and synthesized a series of benzimidazole bearing thiazolidinedione derivatives, and proved remarkable cytotoxicity of these compounds (**254–256)** towards PC-3, HeLa, A549 and HT1080 cancer cell lines with IC_50_ values of 0.096–0.63 µM. In a different study, Compound **257** exhibited potent antiproliferative effect against MFC cells IC_50_ (mean ± SD) value of 25.72 ± 3.95 μM (Wang et al., 2017). Triazole containing 4,5,6,7-tetrabromo-1*H*-benzimidazole derivatives **258–259** bearing carboxyl substituent manifested the most prominent inhibitory effect against protein kinase 2 (CK2) with binding affinity value in the range of 1.96–0.91 µM (Chojnacki et al., 2017). Compounds **260–264** exerted antiproliferative property in MTT assay against five human cancer cell lines, breast (T47D), lung (NCl H-522), liver (HepG2), colon (HCT-15) and ovary (PA-1) with IC_50_ (mean ± SD) value of 7.5 ± 0.3 to 14.6 ± 0.4 μM (Kumar et al., 2018). Upon assessment of antiproliferative property against four cancer cell lines (HeLa, MCF-7, A549 and DU-145 alongside normal HEK-293 cell line), Baig et al. (Baig et al., 2018) enumerated noteworthy IC_50_ (1.08 µM) of derivative **265** against A549 cell line. Compounds **266–268** displayed prominent cytotoxic activity against A549 and MCF-7 cancer cell lines in comparison with standard drug cisplatin with IC_50_ values of 0.03–0.06 μM. The presence of 2,4-dichlorobenzylidene (**266**), 2-nitrobenzylidene (**267**) and 2-methoxybenzylidene moiety (**268**) contributed for notable cytotoxic activity of these compounds (Acar Çevik et al., 2018). The oxetanyl substituted compound **269** exhibited cytotoxicity towards a wide range of cancer cell types, e. g. lung, prostate and ovarian cancers with prominent activity against highly aggressive cancer lines (IC_50_ = 0.9–3.8 µM) (Cheong et al., 2018). Besides, Ibrahim et al. synthesized 2-substituted-5-nitro-benzimidazole derivative **270** as dual inhibitors of c-Met and VEGFR-2 kinases which is important therapeutic target in the treatment of lung (IC_50_ 2.19 ± 0.09 against A549) and colorectal (IC_50_ 10.97 ± 0.09 µM against HCT116) cancers (Ibrahim et al., 2018).

**FIGURE 11:**
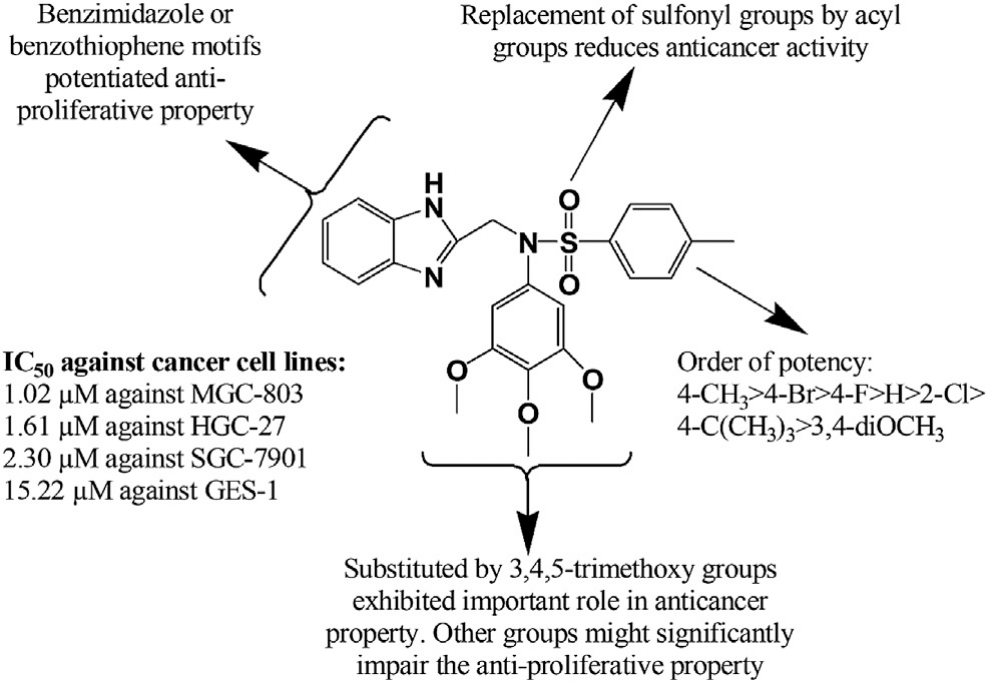
Structure activity relationship (SAR) of benzimidazole derivatives having anticancer potentiality. The figure represents the SAR studies accomplished by Song, J. et al. (2019).

Recently, Djemoui et al. (Djemoui et al., 2020) synthesized several triazole-benzimidazole-chalcone hybrid compounds **271–274** and narrated potential anti-proliferative property of these derivatives against two breast cancer (T47-D and MDA-MB-231) and one prostate cancer cell line (PC3) compared to standard Doxorubicin. It is mentionable here that the chloro substituent **274** at the chalcone ring proliferated the anticancer effects. In a distinct research, a total of 24 new molecules containing benzimidazole group, arene, and alkyl chain-bearing cyclic moieties were synthesized, where the compound **275** impeded the growth of MCF-7 and human ovarian carcinoma (OVCAR-3) cell lines manifesting superior effects to standard cisplatin (IC_50_ (mean ± SD, µM): 8.91 ± 0.07 vs. 11.7 ± 0.12 and 10.76 ± 0.12 vs. 16.04 ± 0.74, respectively) (Hsieh et al., 2019). A copper (II) complex (**276**) of benzimidazole derivatives showed excellent potency at 72 h post treatment against prostate cancer cell line (DU145) with IC_50_ 10 µM (Kacar et al., 2020). Besides, two zinc (II) complexes with 2-[2-(benzimidazol-2-yl)-phenyl]-1-methyl-benzimidazole and 1,2-bis(1-methyl-benzimidazol-2-yl)-benzene exerted both dose and time dependent cytotoxicity against breast cancer cell lines (MB-MDA-231) (Su et al., 2019). In another study (Yılmaz et al., 2019), 18 complexes of zinc (II) and cobalt (II) containing 1-benzyl and 2-phenyl moieties showed *in vitro* potency against human prostate (DU-145) and human ovarian (A-2780) cancer cell lines. Notably, compounds **277–280** at a concentration of 0.1 µM exhibited superior activity against A-2780 cell line in comparison with standard docetaxel. Jian-Song et al. (Jian-Song et al., 2019) synthesized a spectrum of unconventional BZD derivatives and reported their *in vitro* anticancer property, particularly against three genre of cell lines (MGC-803, PC-3, MCF-7). Notably, compound **281** inhibited predominately of all the three cancer cell lines compared to 5 Fluorouracil (IC_50_ (mean ± SD, µM): 1.02 ± 0.03 vs. 6.82 ± 1.17, 3.34 ± 0.09 vs. 18.42 ± 1.73, and 5.40 ± 0.51 vs. 17.11 ± 2.94, respectively). The structural modifications of the compound **281** might significantly influence its anti-proliferative property that has been illustrated in Figure 11. In another study (Suk et al., 2019), a benzimidazole derivative carrying a pyrrolidine side chain (**282)** significantly suppressed sorafenib-resistant cell lines growth in xenograft model by inhibiting the phosphorylation of AKT, p70S6 and the downstream molecule RPS6, has unlocked another milestone in the treatment of hepatocellular carcinoma. Yeong et al. (Yeong et al., 2019) synthesized several new benzimidazole derivatives **283–287** and screened them against sirtuin cancer lines (SIRT1, SIRT2, and SIRT3). Among them, compound **284** elicited significant inhibition of SIRT1-3 compared to tenovin-6 (IC_50_ (mean ± SD, µM): 7.7 ± 1.4 vs. 42.10, 5.6 ± 1.3 vs. 25.6, and 9.8 ± 2.0 vs. 82.65). Moreover, among 37 synthesized molecules, compound **288** exerted the most inhibition of angiogenesis (79%), and HUVEC and HepG2 cell lines (IC_50_: 1.47 and 2.57 mM, respectively), and VEGFR-2 kinase inhibition (Yuan et al., 2019).

Akkoc et al. (Akkoç et al., 2020) synthesized three benzimidazole derivatives **289–291** and reported the most promising anti-breast cancer feature of compound **291** compared to standard cisplatin (IC_50_ (mean ± SD, µM): 1.26 ± 0.85 vs. 5.77 ± 0.40). In another study, Atmaca et al. (Atmaca et al., 2020) disclosed significant cytotoxicity of compound **292** against breast cancer (MCF-7), prostate cancer (DU-145), and lung cancer (H69AR) with IC50 values of 17.8 ± 0.24, 10.2 ± 1.4 and 49.9 ± 0.22 μg/ml, respectively, compared to 5-Fluorouracil. Compounds **293–294** showed stronger anticancer property against HepG2 (IC_50_: 26.62 and 20.29, respectively) and DLD-1 cells (IC_50_: 21.29 and 19.23 µM, respectively) than cisplatin (IC_50_: 30.38 and 60.79 µM) (Caymaz et al., 2020). Particularly, compound **295–296** demonstrated potent anti-breast cancer effect by obstructing MCF-7 cell growth compared to standard cisplatin (IC_50_: 0.016 ± 0.001 and 0.018 ± 0.001 vs. 0.020 ± 0.009 µM). Besides, compound **295** displayed efficiency for impeding estrogen-dependent breast cancer by inhibiting aromatase enzyme with IC_50_ 0.032 ± 0.042 µM, compared to IC_50_ 0.024 ± 0.001 µM for letrozole (Acar Çevik et al., 2020). Furthermore, compound **297** blocked neddylation process with superior anticancer property compared to candesartan cilexetil (IC_50_ 5.51 vs 16.43 mM) (Chen et al., 2021). Notably, compound **298** displayed excellent effect in the treatment of lung cancer by inhibiting A-549 and NCI-H460 cell lines growth with IC_50_ level of 0.63 ± 0.21 μM and 0.99 ± 0.01 μM, respectively, compared to 5-Fluorouracil (IC_50_ (μM): 1.69 ± 0.90 and 3.20 ± 0.50, respectively). Apart from, the compound **298** significantly suppressed the breast cancer cell lines MCF-7 and MDA-MB-23 with IC_50_ (μM) values, comparable to 5-Fluorouracil (1.3 ± 0.18 vs. 2.80 ± 0.12, and 0.94 ± 0.02 vs. 0.79 ± 0.09, respectively) (Sridhar Goud et al., 2020).

Meguid et al. (El-Meguid et al., 2020) synthesized an array of novel 6-benzoyl benzimidazole derivatives where most of the compounds exhibited promising anticancer activity with safety profile. Remarkably, compounds **299–300** exhibited superior inhibition of EGFR, HER2, PDGFR-β and VEGFR2, in comparison to erlotinib that opened several promising fighting tools against cervical cancer. In a distinct study, compounds **301–304** strongly prevented breast cancer cell lines manifesting IC_50_ values of 5.70 9.55, 5.58 and 6.84 μg/ml, respectively compared to standard doxurubucin (IC_50_ at 4.17 μg/ml) (Nashaat et al., 2020). Furthermore, Sireesha et al. (Sireesha et al., 2021) synthesized a library of hybrid *β*-carbolines **305–311** that were found to be effective against various cancer cell lines where compounds **306–307** exerted higher *in vitro* efficacy than the reference etoposide to prevent breast (IC_50_ against MCF-7: 0.092 ± 0.001 and 0.81 ± 0.062, vs. 2.11 ± 0.024), lung (IC_50_ against A549: 0.72 ± 0.042 and 1.90 ± 0.88, vs. 3.08 ± 0.135), colon (IC_50_ against Colo-205: 0.34 ± 0.071 and 0.41 ± 0.12, vs. 0.13 ± 0.017), and ovarian (IC_50_ against A2780: 1.23 ± 0.55 and 1.80 ± 0.59, vs. 1.31 ± 0.27) cancers. Similarly, Srour et al. (Srour et al., 2020) synthesized a series of benzimidazole derivatives **312–318** and reported promising anticancer potential of **312–316** against breast cancer **(**IC_50_ against MCF-7: 5.96–11.91 μM, vs. IC_50_ of erlotinib; 4.15 μM). Besides, compounds **312, 314, 316, 317** and **318** showed significant cytotoxicity against epidermal growth factor receptor tyrosine kinase with IC_50_ values of 71.67–152.59 nM compared to IC_50_ of standard erlotinib 152.59 nM. Finally, Yamani et al. (Yamani et al., 2021) applied scaffolds hybridization technique to formulate a total of 24 pyrazole-benzimidazole derivatives for blocking fibroblast growth factor receptors (FGFRs). Amongst the derivatives, compound **319** selectively inhibited FGFR (1–4) with IC_50_ values of 0.75, 0.50, 3.05, and 87.90 nM, respectively. Due to acceptable safety and pharmacokinetic profiles along with *in vivo* anti-tumor potency, the compound **319** is now undergoing with an open-label, multicenter, dose-escalation phase I clinical trial for assessing the safety and tolerability against the adults patients with bladder, gastric, and squamous cell lung cancers (NCT04149691) (Yamani et al., 2021).

### Antitubercular Activity

**FIGURE 12:**
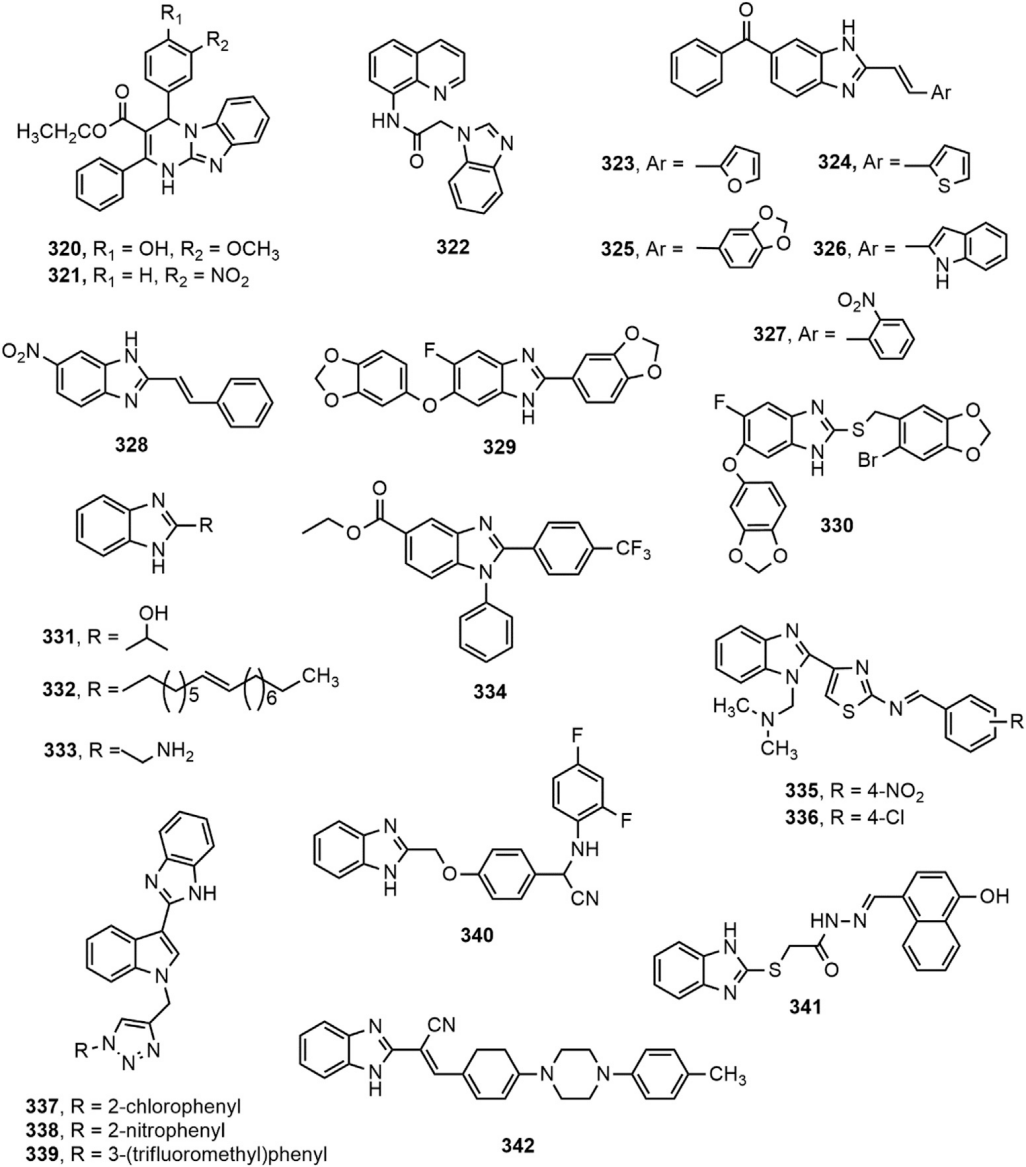
Benzimidazole derivatives with antitubercular activity.

Compounds containing heterocyclic moieties, such as pyrrole, imidazole and benzimidazole have been reported to demonstrate excellent antitubercular properties (Wang et al., 2015). Benzimidazole scaffold has been on target of the scientists for producing novel antitubercular agents. Different benzimidazole derivatives with antitubercular property are shown in Figure 12.

Warekar et al. (Warekar et al., 2016) synthesized a series of 4-(4-nitro-phenyl)-2-phenyl-1,4-dihydro-benzo[4,5]imidazo[1,2-a]pyrimidine-3-carboxylic acid ethyl ester derivatives and reported that compounds **320–321** appeared to be the most promising antitubercular agents among the series with minimum inhibitory concentration (MIC) value of 25 μg/ml against *Mycobacterium tuberculosis* H37Rv strain. Compound **322** also displayed good activity against same strain under aerobic conditions, as indicated by its IC_90_ and MIC values (77 µg/ml and >100 μM, respectively) (Mantu et al., 2016). In a different study (Anguru et al., 2017), compounds **323–328** exhibited good activity against *M. tuberculosis* H37Rv strain while compound **325** emerged as the most promising agent with MIC value of 16 μg/ml.

A number of substituted fluorobenzimidazole derivatives (**329–330**) were synthesized and evaluated for *in vitro* antimycobacterial property against pathogenic *M. tuberculosis* H37Rv strain (ATCC 27294) using MABA method. Compounds **329–330** exhibited notable antitubercular activity against H37Rv strain and their activity was contributed by the incorporation of methylenedioxyphenyl moiety at 2- and 6-position of benzimidazole ring (Nandha et al., 2017). Harika et al. (Harika et al., 2017) synthesized a series of 2-substituted benzimidazole derivatives (**331–333**) using condensation of *o-*phenylenediamine with different aliphatic, aromatic, fatty acids, and amino acids, and depicted remarkable antitubercular property against *M. tuberculosis* H37Rv strain compared to reference drugs pyrazinamide, streptomycin and ciprofloxacin. In addition, Yeong et al. (Yeong et al., 2017) designed two series of benzimidazole derivatives among which compound **334** having trifluoromethyl group displayed antimycobacterial effect against both *M. tuberculosis* H37Rv strain and the drug-resistant-tuberculosis strain. Compounds **335–336**, synthesized by Prasad and Sundararajan, exhibited notable antitubercular activity against *M. tuberculosis* H37Rv strain with MIC value of 3.9 µg/ml compared to the standard isoniazid (Prasad and Sundararajan, 2017).

**FIGURE 13:**
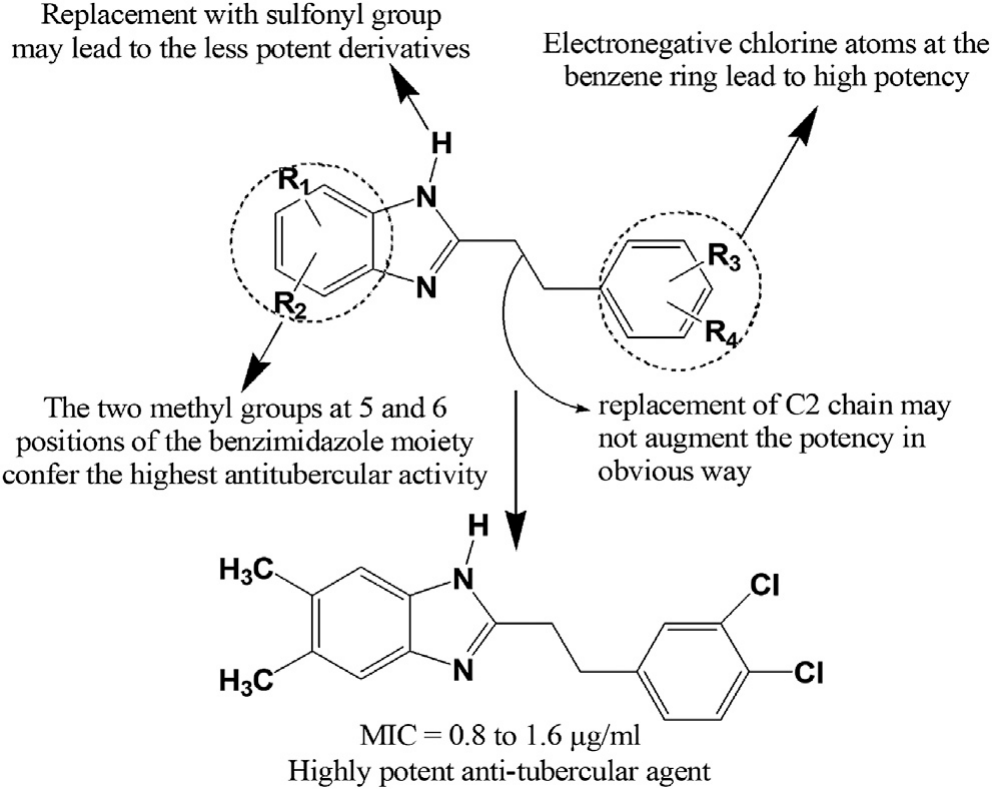
Structure activity relationship (SAR) of benzimidazole derivatives having anti-tubercular effects. The figure represents the SAR studies accomplished by Gobis et al. (2015).

Recently, Ashok et al. (Ashok et al., 2018) synthesized a series of indole-benzimidazole-based 1,2,3-triazole hybrids (**337–339**) by conventional and microwave-assisted methods and evaluated for *in vitro* antitubercular activity against *M. tuberculosis* H37Rv strain. The derivatives **337–339** showed prominent antitubercular activity with MIC values in the range of 3.125–6.25 μg/ml. Compound **338** was the most potent among all (MIC = 3.125 μg/ml) which was likely due to the presence of nitro group at ortho position of phenyl ring. Compound **340** displayed notable activity (MIC = 0.05 μg/ml) and emerged as a promising antitubercular agent among the reported series (Shaikh et al., 2018). Compound **341** showing 67.56, 53.45, and 47.56% inhibition against mycobacterial enzymes isocitrate lyase, pantothenate synthetase and chorismate mutase, respectively appeared to be the most potent antitubercular agent among the series (Yadav et al., 2018). Very recently, compound **342** exerted excellent potency against *M. tuberculosis* H37Rv with IC_50_ value of 0.78 mg/ml compared to standard ethambutol (IC_50_ 1.56 mg/ml) (Sirim et al., 2020). Gobis et al. (Gobis et al., 2015) have prepared a series of compounds containing novel 2-(2-phenalkyl)-1*H*-benzo[d]imidazole where the compound bearing methyl groups at the benzimidazole system and phenethyl substituent at the C-2 position with electronegative chlorine atom at the phenyl ring exhibited prominent tuberculostatic property against *Mycobacterium tuberculosis* strains with MIC values ranging from 0.8 to 1.6 μg/ml (Figure 13).

### Antiprotozoal Activity

**FIGURE 14:**
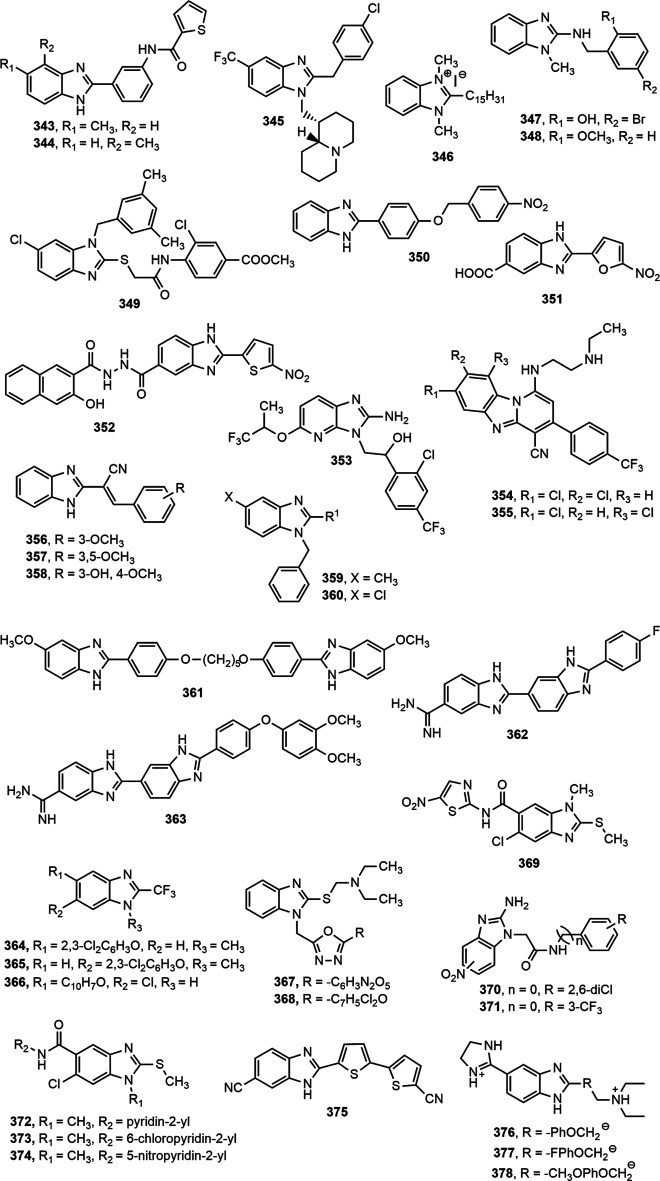
Benzimidazole derivatives with antiprotozoal activity.

The exploration of benzimidazole nucleus to discover new structural features required for the optimization of novel antiprotozoal agents is of utmost importance. Benzimidazole derivatives with antileishmanial, antimalarial, and antiprotozoal activities against different species are shown in Figure 14.

#### Benzimidazole Against *Leishmania* Species

Keurulainen et al. (Keurulainen et al., 2015) synthesized several 2-arylbenzimidazole derivatives and proclaimed extensive inhibitory property of compounds **343–344** against axenic amastigotes of *Leishmania donovani*. Tonelli and co-workers (Tonelli et al., 2018) reported *in vitro* antileishmanial activity of derivatives **345–346** with IC_50_ values of 3.70 and 0.19 µM, respectively against *L. tropica*, and 4.76 and 0.64 µM, respectively against *L. infantum*. In another research, a total of 28 *N*-benzyl-1*H*-benzimidazol-2-amine derivatives, where compounds **347–348** showed significant (*p* < 0.05) antileishmanial activity against the amastigote of *L. mexicana* and *L. braziliensis* with IC_50_ values of 2.62 and 3.21 µM, respectively, and their activity was 5.8 and 4.8 times better than standard miltefosine (IC_50_ = 15.34 µM) (Nieto-Meneses et al., 2018). Upon evaluation of *in vitro* antileishmanial activity against intracellular amastigotes of *L. infantum*, compound **349** displayed promising result with IC_50_ value of 6.8 μM. However, the compound possessed some degree of cytotoxicity with CC_50_ = 8.0 and 32.0 μM against primary peritoneal mouse macrophages PMM and human fetal lung fibroblasts MCR-5, respectively (De Luca et al., 2018). Recently, compounds **350** exerted moderate antileishmanial effect by inhibiting *L. donovani* with IC_50_ value of 68 ± 2.8 μM compared to standard miltefosine (IC_50_ = 12 ± 0 μM) (Kapil et al., 2019).

#### Benzimidazole Against Malaria

Among the four *Plasmodium* species responsible for human malaria, *P. falciparum* has already started to show resistance to available antimalarial drugs, thus causing the urgency to develop drugs with novel drug targets and new mechanism of action (Singh et al., 2012). In search for compounds with comparable activity to chloroquine, Camacho et al. (Camacho et al., 2011) developed a series of benzimidazole-5-carbohydrazide derivatives and reported that the compounds **351–352** showed prominent *in vivo* antimalarial potential against rodent *P. berghei* and appeared to be as effective as chloroquine. Based on structure-activity relationship studies and pharmacokinetics optimization, compound **353** was found to display noticeable efficacy in the humanized *P. falciparum* mouse model of malaria (*Pf*/SCID model) with ED_90_ value of 28.6 mg/kg. The attachment of the neutral hydrophobic group at position 6 of the benzimidazole moiety enhanced this excellent anti-malarial property (Hameed et al., 2014). Singh et al. (Singh K. et al., 2017) reported a series of pyrido[1,2-*a*]benzimidazole (PBI) antimalarial agents having *in vitro* anti-plasmodial activity with IC_50_ values of 0.02–0.95 μM against *Pf* NF54 strain, and 0.02–1.07 μM against multidrug-resistant *Pf* K1 strain of *P. falciparum*. Among which, compounds **354–355** exhibited most prominent *in vivo* efficacy in mouse *P. berghei* model due to the presence of chlorine group at C-7, C-8 and C-9 position of benzimidazole moiety. In another study, compounds **356–358** displayed excellent activity with IC_50_ values of 0.69, 1.60 and 1.61 μM, respectively against chloroquine-sensitive 3D7 strain compared to standard chloroquine (IC_50_ = 1.53 µM) (Sharma et al., 2018). Recently, compounds **359–360** were synthesized as highly potent antimalarial agents having IC_50_ values of 0.098 and 0.062 μM, respectively against NF54 strain of *P. falciparum* (Mueller et al., 2020)*.*

#### Benzimidazole Against Different Protozoa

Torres-Gómez et al. (Torres-Gómez et al., 2008) synthesized a library of benzimidazole-pentamidine hybrids and evaluated them for antiprotozoal activity against *T. vaginalis*, *E. histolytica*, *G. lamblia*, *L. Mexicana* and *P. berghei*. Compound **361** was found to be the most potent from the series, showing 3- and 9- times more activity than standards metronidazole and pentamidine, respectively. Alp et al. (Alp et al., 2009) synthesized several 2’-arylsubstituted-1*H*,1’*H*-[2,5’]-bisbenzimidazolyl-5-carboxamidine derivatives and found promising antiparasitic activity of compounds **362** and **363** against *P. falciparum, L. donovani, T. brucei rhodesiense* and *Trypanosoma cruzi.* The presence of 4-fluorophenyl (**362**) and 4-(3,4-dimethoxyphenoxy)phenyl groups (**363**) at the C-2’ position of amidinobisbenzimidazole moiety contributed for the antiparasitic activity. Among a library of 2-(trifluoromethyl)-1*H-*benzimidazoles, compounds **364–366** displayed the most prominent *in vitro* antiparasitic activity against *E. histolytica*, *G. intestinalis*, *T. vaginalis* and *T. spiralis* (Hernández-Luis et al., 2010). Compounds **367–368** manifested notable activity against *Paramecium caudatum* and *Vorticella campanula* compared to standard metronidazole (Maske et al., 2012).

**FIGURE 15:**
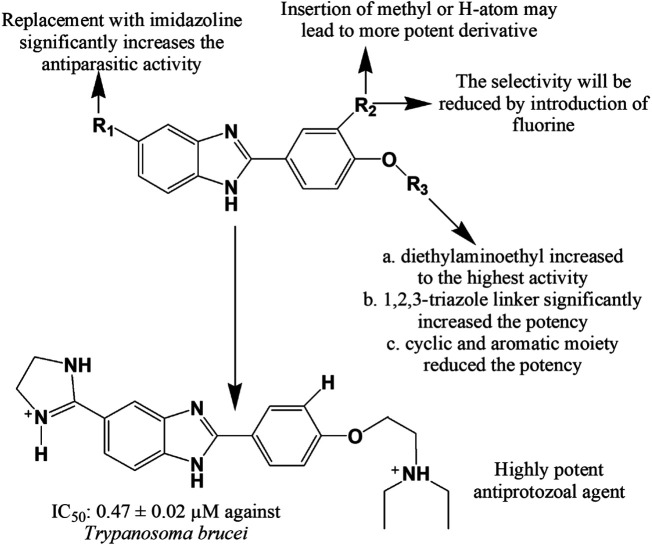
Structure activity relationship (SAR) of benzimidazole derivatives effective against *Trypanosoma brucei*. The figure represents the SAR studies accomplished by Popov et al. (2020).

Matadamas-Martínez et al. (Matadamas-Martínez et al., 2016) designed and synthesized a novel nitazoxanide and N-methyl-1*H*-benzimidazole hybrid molecule **369** which displayed better activity with IC_50_ value of 0.010 μM than that of standards nitazoxanide, albendazole and metronidazole against *G. intestinalis* (IC_50_ = 0.015, 0.037 and 1.224 μM, respectively). Hernández-Núñez et al. (Hernández-Núñez et al., 2017) prepared a series of 2-(2-amino-5(6)-nitro-1*H*-benzimidazol-1-yl)-*N*-arylacetamide analogues and illustrated 7-fold more potency of compound **370** than the standard benznidazole against *G. intestinalis* with an IC_50_ of 3.95 µM, and 4-fold more activity of compounds **370–371** against *T. vaginalis* in comparison with benznidazole. Flores-Carrillo and co-workers (Flores-Carrillo et al., 2017) prepared a library of twelve 2-(methylthio)-1*H*-benzimidazole-5-carboxamide derivatives and investigated their *in vitro* antiparasitic activity against *G. intestinalis*, *E. histolytica* and *T. vaginalis*. Compounds **372** and **373** showed notable effect against *T. vaginalis* and *G. intestinalis*, respectively in comparison with standards albendazole and metronidazole, and compound **374** emerged as a broad-spectrum antiprotozoal agent with activity against all three tested protozoans. Farahat et al. (Farahat et al., 2018) synthesized a series of benzimidazole bichalcophene diamidine derivatives. Compound **375** showed prominent antiparasitic activity towards mice model infected with *T. brucei rhodesiense* at a dose of 4 × 5 mg/kg i.p. and was found to be more potent than pentamidine, the usual drug of choice to treat African sleeping sickness. Similarly, compounds **376–378** exerted superior efficacy against *T. brucei* in the treatment of human African trypanosomiasis with IC_50_ (mean ± SEM) values of 0.47 ± 0.02, 3.67 ± 0.30, and 0.71 ± 0.22, respectively compared to standard nifurtimox (IC_50_ = 2.0 ± 0.2). The presence of diethylaminoethyl group substantially augmented the antitrypanosomal property of the compounds (**376–378**). Unsubstituted or methyl substituted aromatic rings or inclusion of an imidazole ring at C-5 also potentiated the activity (Figure 15) (Popov et al., 2020).

### Antihypertensive Activity

**FIGURE 16:**
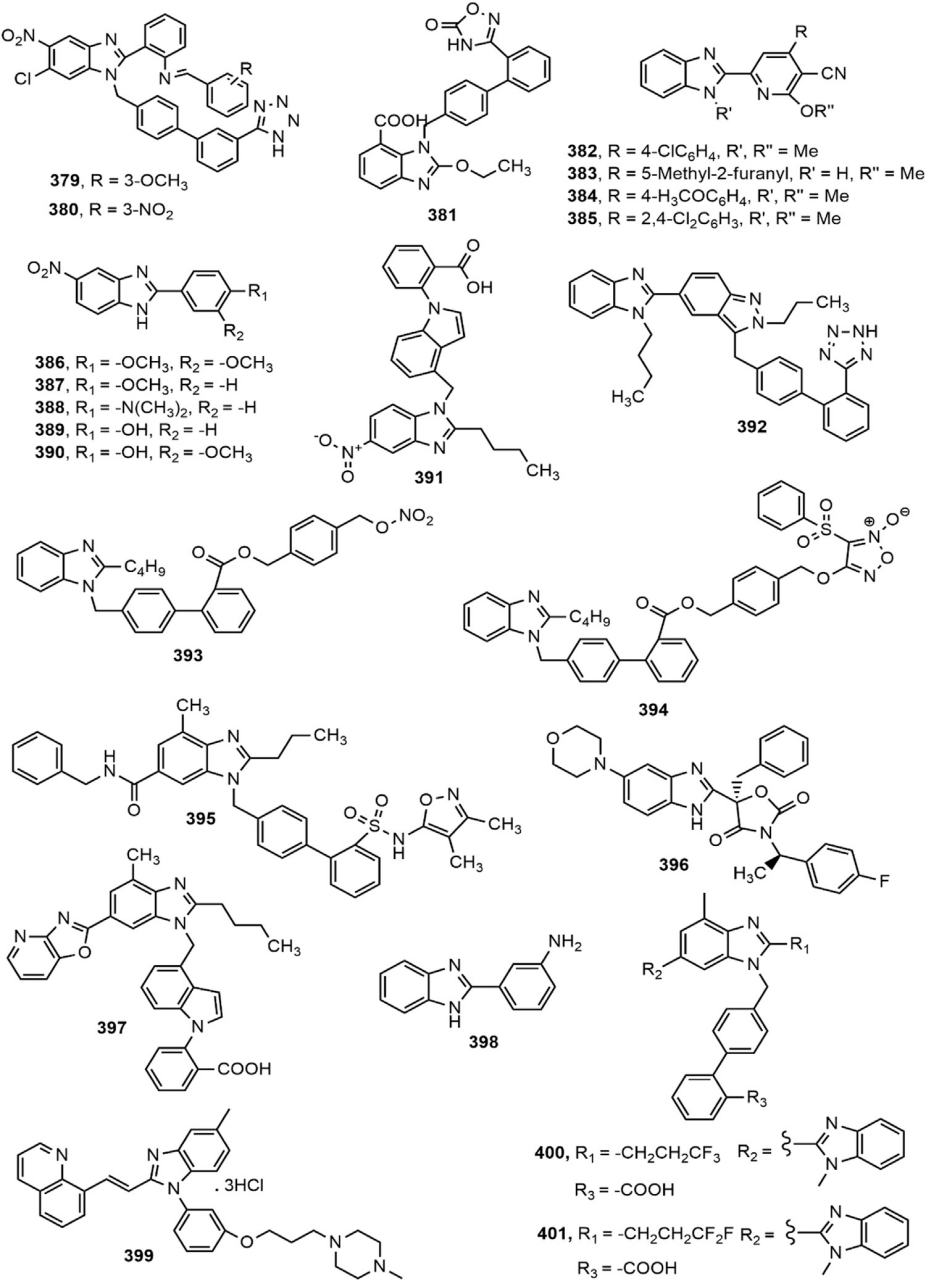
Benzimidazole derivatives with antihypertensive activity.

A number of marketed antihypertensive drugs comprise benzimidazole moiety, Candesartan cilexetil and Telmisartan are two major examples (Figure 2). Categorically they are the antagonists of angiotensin II receptor playing important role in managing hypertension (Keri et al., 2015). In recent years, a number of scientists have conducted research to prepare benzimidazole based novel antihypertensive agents which provided similar or even better efficacy than the conventional types of antihypertensive drugs. Different benzimidazole derivatives with antihypertensive activity are shown in Figure 16.

Sharma et al. (Sharma et al., 2010) synthesized a series of substituted benzimidazole derivatives and evaluated for their property as angiotensin II receptor antagonists or sartans using invasive (direct) method in Wister rats. Compounds **379** and **380** appeared to be the most prominent antihypertensive agents from the series compared to standard losartan. Kusumoto et al. (Kusumoto et al., 2011) reported about a novel, long-lasting and potent AT_1_ blocker azilsartan medoxomil and its active metabolite azilsartan (**381**) and investigated its pharmacological profile in rat and dog models. Oral administration of 0.1–1 mg/kg azilsartan medoxomil in spontaneously hypertensive rats (SHRs) and renal hypertensive dogs demonstrated better effect in reduction of blood pressure at all doses compared to standard drug olmesartan medoxomil. Abou-Seri et al. (Abou-Seri et al., 2011) synthesized a series of 2-alkoxy-4-aryl-6-(1*H*-benzimidazol-2-yl)-3-pyridinecarbonitrile derivatives. All compounds in the series displayed significant vasodilation properties. Compounds **382–385** showed most prominent activity (IC_50_ = 0.145, 0.202, 0.210, and 0.214 mM, respectively) compared to standard prazosin hydrochloride (IC_50_ = 0.487 mM).

Datani et al. (Datani et al., 2012) synthesized a series of twenty novel 5-nitro benzimidazole analogues and screened them for *ex vivo* vasorelaxant property in rat aorta rings pre-contracted with phenylephrine. Compounds **386–390** exhibited prominent vasorelaxant activity with EC_50_ value less than 30 μM. Among a new set of 5-nitro benzimidazole derivatives, compound **391** emerged as the most active agent against AT_1_ with IC_50_ value of 1.03 ± 0.26 nM (Zhu et al., 2014). The presence of butyl chain on 2-position of benzimidazole moiety (**391**) helped the derivative to interact and bind tightly with lipophilic pocket of the receptor. Several benzimidazole derivatives containing indazole moiety were synthesized by Lamotte et al. (Lamotte et al., 2014) among which compound **392** displayed potent AT_1_ receptor antagonism as indicated by IC_50_ value (0.006 mM).

Two series of nitric oxide (NO) releasing benzimidazole derivatives were synthesized by coupling benzimidazole biphenyl skeleton with nitro ester and furoxan NO-donor moieties, where compounds **393–394** were reported to possess comparable activity to positive control losartan (Zhang et al., 2015). Hao et al. (Hao et al., 2015) designed and synthesized a series of 4′-[(benzimidazol-1-yl)methyl]biphenyl-2-sulphonamide derivatives and reported that compound **395** was found to be the most potent AT_1_ and Endothelin ET_A_ receptor antagonist with IC_50_ values 28 and 10 nM, respectively. Upon the identification of a new series of benzimidazole oxazolidinediones as mineralocorticoid receptor (MR) antagonists by high-throughput screening, compound **396** showed similar efficacy as standard drug spironolactone at a dose of 100 mg/kg (p.o.) in rat natriuresis model (Yang et al., 2015).

Bao et al. (Bao et al., 2017) synthesized a series of benzimidazole derivatives which reduced blood pressure in dose-dependent manner in spontaneously hypertensive rats. Among the series, compound **397** exhibited long-lasting efficiency in decreasing blood pressure, with a maximal lowered response of 35.82 ± 6.20 mmHg at 5 mg/kg and 55.98 ± 4.74 mmHg at 10 mg/kg. The compound also showed potent affinity towards AT_1_ receptor compared to standard telmisartan with IC_50_ value of 1.13 ± 1.68 nM. In a separate study, Khan et al. (Khan et al., 2018) synthesized a series of 2-phenyl substituted benzimidazoles and assessed antihypertensive activity of these derivatives by using tail cuff method and confirmed excellent antihypertensive property of compound **398** in spontaneously hypertensive rats compared to standard losartan. A very recent report (Yang et al., 2020) ascertained the promising pulmonary hypotensive effect of compound **399** with excellent pharmacokinetic profile in comparison with tadalafil. Finally, compounds **400–401** showed superior inhibition of AT1 receptor (IC_50_ (mean ± SEM): 0.8 ± 0.1 and 2.3 ± 0.7, respectively) than the both standard losartan and telmisartan (Wu et al., 2020).

### Antidiabetic Activity

**FIGURE 17:**
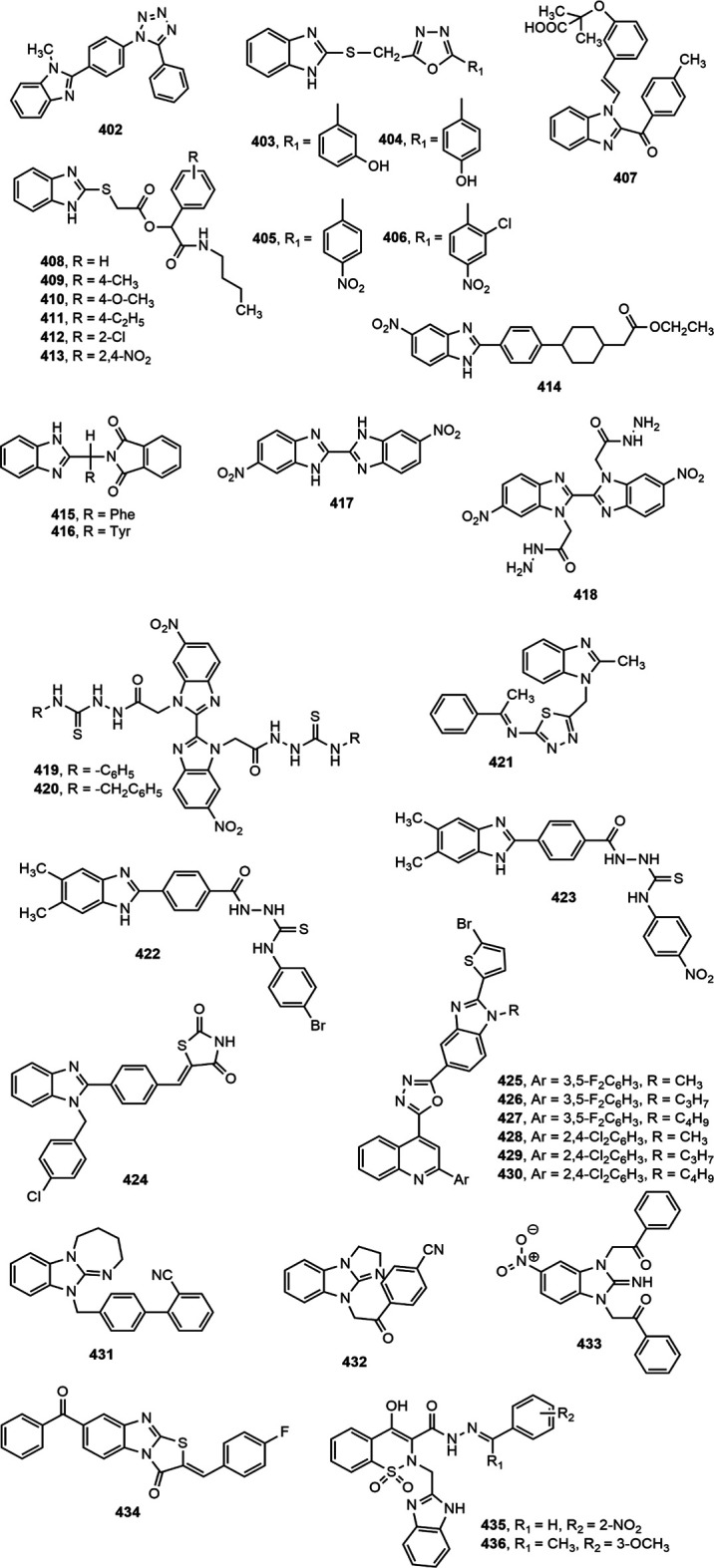
Benzimidazole derivatives with antidiabetic activity.

Several benzimidazole based compounds have displayed promising antidiabetic activity by acting as targets of varied stages of carbohydrate metabolism and some of them have been marketed for the treatment of type 2 diabetes. The structure of benzimidazole derivatives with antidiabetic activity reported within recent years are shown in Figure 17.

Several 2-(pyridine-2-yl)-1*H*-benzimidazole derivatives were synthesized by Ishikawa et al. (Ishikawa et al., 2009) among which compound **402** emerged as the most potent and metabolically stable glucokinase activator. Besides, the compound proved its oral glucose lowering efficacy in rat oral glucose tolerance test (OGTT) model. A new series of 4-thiazolidinones and 1,3,4-oxadiazoles bearing 2-mercapto benzimidazole moiety were prepared by Shingalapur et al. (Shingalapur et al., 2010) among which compounds **403–406** produced notable results in OGTT model. All the potent derivatives contained hydroxyl group which might have contributed for their antidiabetic property. Some 2-benzoyl benzimidazole derivatives were assessed for antidiabetic and lipid-lowering effects (Ushiroda et al., 2011). Compound **407** appeared to be an effective peroxisome proliferator-activated receptor (PPARγ) partial agonist. Shaikh et al. (Shaikh et al., 2012) reported one-pot synthesis of carbonyl-amide linkage based benzimidazole derivatives (**408–413**) *via* Passerini reaction and demonstrated their antidiabetic potential using rat OGTT model compared to the standard drug glibenclamide.

Diacylglycerol O-acyltransferase-1 (DGAT-1) is an enzyme which catalyzes the formation of triglycerides from diacylglycerol and Acyl-CoA and thereby plays an important role in intestinal fat absorption. DGAT-1 has emerged as an important therapeutic target for the management of different metabolic disorders, such as obesity and diabetes (Birch et al., 2010). Kwak et al. (Kwak et al., 2013) prepared some benzimidazole derivatives as inhibitors of DGAT-1 which contained a phenylcyclohexyl acetic acid group in benzimidazole moiety. Among the derivatives, compound **414** displayed potent *in vivo* antidiabetic potential in a 4-week study with diet-induced obesity (DIO) mouse model.

The α-glucosidase inhibitors are an important class of antidiabetic agents because of their property to reduce the postprandial glucose level in type-2 diabetic patients (Özil et al., 2016). Mobinikhaledi et al. (Mobinikhaledi et al., 2015) synthesized several new benzimidazole derivatives from amino acids in the presence of phosphorus oxychloride (POCl_3_). Compounds **415** and **416**, upon evaluation of yeast and rat intestinal α-glucosidases inhibitory effect produced most notable results. The IC_50_ values for compound **416** against yeast and rat intestinal α-glucosidases were reported as 9.1 and 36.7 μM, respectively and thus it appeared to be the most potent benzimidazole among the series. Özil and co-workers (Özil et al., 2016) prepared a series of bis-benzimidazole derivatives by the reaction of *o*-phenylenediamine and 4-nitro-*o*-phenylenediamine with oxalic acid using both conventional and microwave techniques. Compounds **417–420** demonstrated prominent α-glucosidase inhibition with IC_50_ values of 0.54 ± 0.01, 0.44 ± 0.04, 1.24 ± 0.05 and 0.49 ± 0.01 µM, respectively compared to standard acarbose (IC_50_ 13.34 ± 1.26 µM).

Some novel 1,3,4-thiadiazole substituted 2-methyl benzimidazole derivatives were synthesized by conventional methods among which compound **421** exhibited significant *in vitro* antidiabetic property (Nair et al., 2016). A series of hybrid benzimidazole-thiourea derivatives were synthesized by Zawawi et al. (Zawawi et al., 2017) and evaluated for α-glucosidases inhibitory potential. Compounds **422–423** displayed significant inhibitory properties with IC_50_ values of 50.57 ± 0.81 and 35.83 ± 0.66 µM, respectively. Besides, Singh et al. (Singh et al., 2018) synthesized a library of *N*-substituted-benzimidazolyl linked *para* substituted benzylidene derivatives. Compound **424** bearing 2,4-thiazolidinedione group at 4-position of phenyl ring exhibited pronounced *in vitro* α-amylase and α-glucosidase inhibitory properties (IC_50_ = 0.54 ± 0.01 µM) and appeared as a promising antidiabetic agent from the series.

Recently, Bharadwaj et al. (Bharadwaj et al., 2018) have developed several novel benzimidazole derivatives **(425–430)** and reported excellent antidiabetic activity by applying α-glucosidase inhibitory method with a range of IC_50_ = 0.66 ± 0.05 to 3.79 ± 0.46 μg/ml compared to standard IC_50_ value of acarbose (1460.28 ± 244.365). Besides, two potent AMP-activated protein kinase (AMPK) activators (**431–432**) with multi-target antidiabetic property were reported against LPS-activated murine peritoneal macrophages (Babkov et al., 2019). In another study, two molecules, 1,3-disubstituted-benzimidazole-2-imine (**433**) and 1,3-thiazolo[3,2-*a*]benzimidazolone derivative (**434**), exerted dual effects against dipeptidyl peptidase-IV (DPP-IV) and xanthine oxidase (XO) enzymes with IC_50_ values less than 200 μM (Tomovic et al., 2020). Finally, Kanwal et al. (Kanwal et al., 2020) prepared a library of benzimidazole-benzothiazine hybrid molecules by Gabriel–Colman rearrangement of methyl 2-(1,1-dioxido-3-oxobenzo[*d*]isothiazol-2(3*H*)-yl) acetate, and reported that compounds **435–436** exhibited potential antidiabetic property by inhibiting Ecto-nucleotide pyrophosphatases/phosphodiesterases 1 (ENPP1) 1 and -3.

### Anticoagulant Activity

**FIGURE 18:**
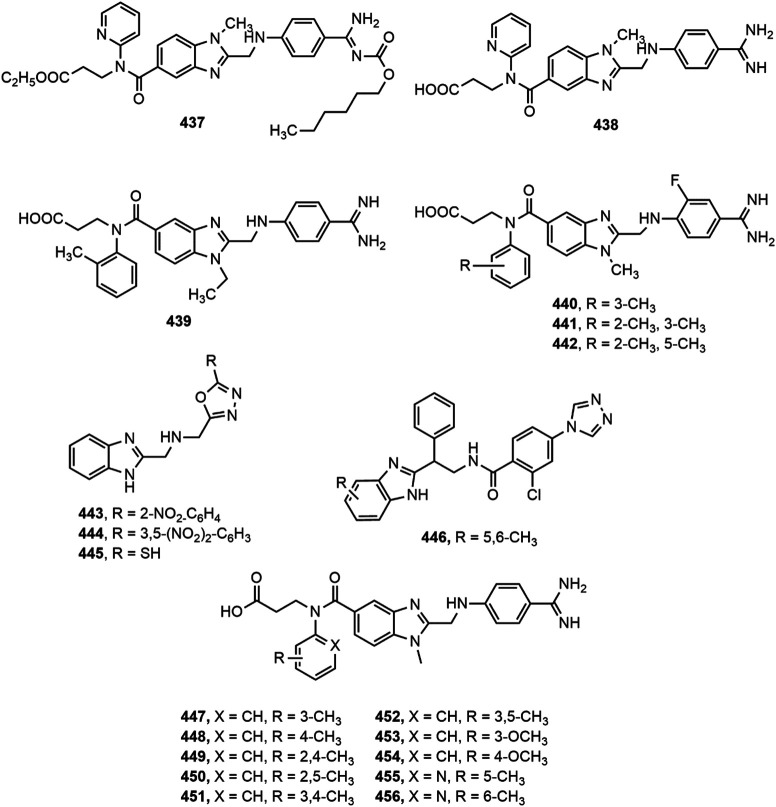
Benzimidazole derivatives with anticoagulant activity.

Different substituted benzimidazole derivatives have been explored for several years for their anticoagulant activity and potential use in clinical practice. Benzimidazole derivatives acting as anticoagulants are shown in Figure 18.

Thrombin (fIIa) is a multifunctional serine protease responsible for the proteolytic cleavage of fibrinogen. Inhibition of thrombin is a crucial mechanism for inhibition of coagulation. Benzimidazole moiety serves as a suitable template for placing a wide range of substituents required for interaction with thrombin (Hauel et al., 2002). Hauel et al. (Hauel et al., 2002) designed and synthesized a series of new benzimidazole derivatives bearing structural similarity to α-NAPAP (*N*-alpha-(2-naphthylsulfonylglycyl)-4-amidinophenylalanine piperidide), a benzamidine-based powerful inhibitor of thrombin, trypsin and other serine proteases. With the addition of ethyl ester and hexyloxycarbonyl carbamide hydrophobic side chains, improved pharmacokinetic profile was obtained leading to the invention of orally absorbed prodrug, **437** (Dabigatran etexilate). The prodrug **437** reached clinical trials and its active form **438** (Dabigatran) was discovered with excellent thrombin inhibitory potency and tolerability.

Recently, Ren et al. (Ren et al., 2016) designed a series of benzimidazole derivatives and tested them for thrombin inhibitory effects. Compound **439** with IC_50_ value of 3.11 ± 0.21 nM appeared to be a potent thrombin inhibitor exhibiting better activity than the standard argatroban (IC_50_ 9.88 ± 2.26 nM). A series of 1,2,5-trisubstituted benzimidazole fluorinated derivatives (**440–442**) were also evaluated for *in vitro* inhibitory activity against thrombin (Yang et al., 2016). Compounds **440–442** with IC_50_ values of 2.26 ± 0.38, 1.54 ± 0.09 and 3.35 ± 0.87 nmol/L, respectively showed improved result compared to argatroban (IC_50_ 9.88 ± 2.26 nmol/L), thus showing their potential as thrombin inhibitors. A library of benzimidazole derived 1,3,4-oxadiazole derivatives (**443–445**) were assessed for *ex vivo* anticoagulant activity by determining the effect of compounds in increasing prothrombin time (PT) and activated partial thromboplastin time (aPTT) (Vishwanathan and Gurupadayya, 2015). Compounds **443–445** displayed significant increase in PT (32 ± 0.7, 36 ± 0.5 and 41 ± 0.4 s, respectively) compared to standard drug acenocoumarol (48 ± 0.5 s). The compounds, however, caused a slight increase in aPTT in comparison with the reference drug, unfractionated heparin (500 IU/kg).

Factor IXa (fIXa), an important coagulation factor, is a useful target for developing potent and selective antithrombotic agents. A research involving pharmacophore modelling of a new series of benzimidazole analogues presented the chemical features necessary for designing fIXa inhibitors and showed that benzimidazole derivatives have the potential to be developed into effective antithrombotic agents (Kumbhar et al., 2017). Compound **446** was found to be the most active compound from the series, indicated by fIXa binding affinity (K_i_) value of 0.016 µM.

Recently, Zhang et al. (Zhang et al., 2020) designed and synthesized ten novel dabigatran derivatives **(447–456)** with high docking score. The study uncovered that all the compounds showed more than 50% *in vitro* thrombin inhibitory property at 1 mg/ml concentration, where the IC_50_ values of compounds **447, 450** and **456** were 1.92, 2.17 and 1.54 nM, respectively, comparable to the IC_50_ value of positive control dabigatran (1.20 nM). The derivatives **425** and **428,** previously reported in this review for antidiabetic property, exerted anticoagulant activity by augmenting the clotting duration. However, only compound **425** exhibited excellent inhibition (93.4%) of epinephrine-induced platelet aggregation (Bharadwaj et al., 2018).

### Anticonvulsant Activity

**FIGURE 19:**
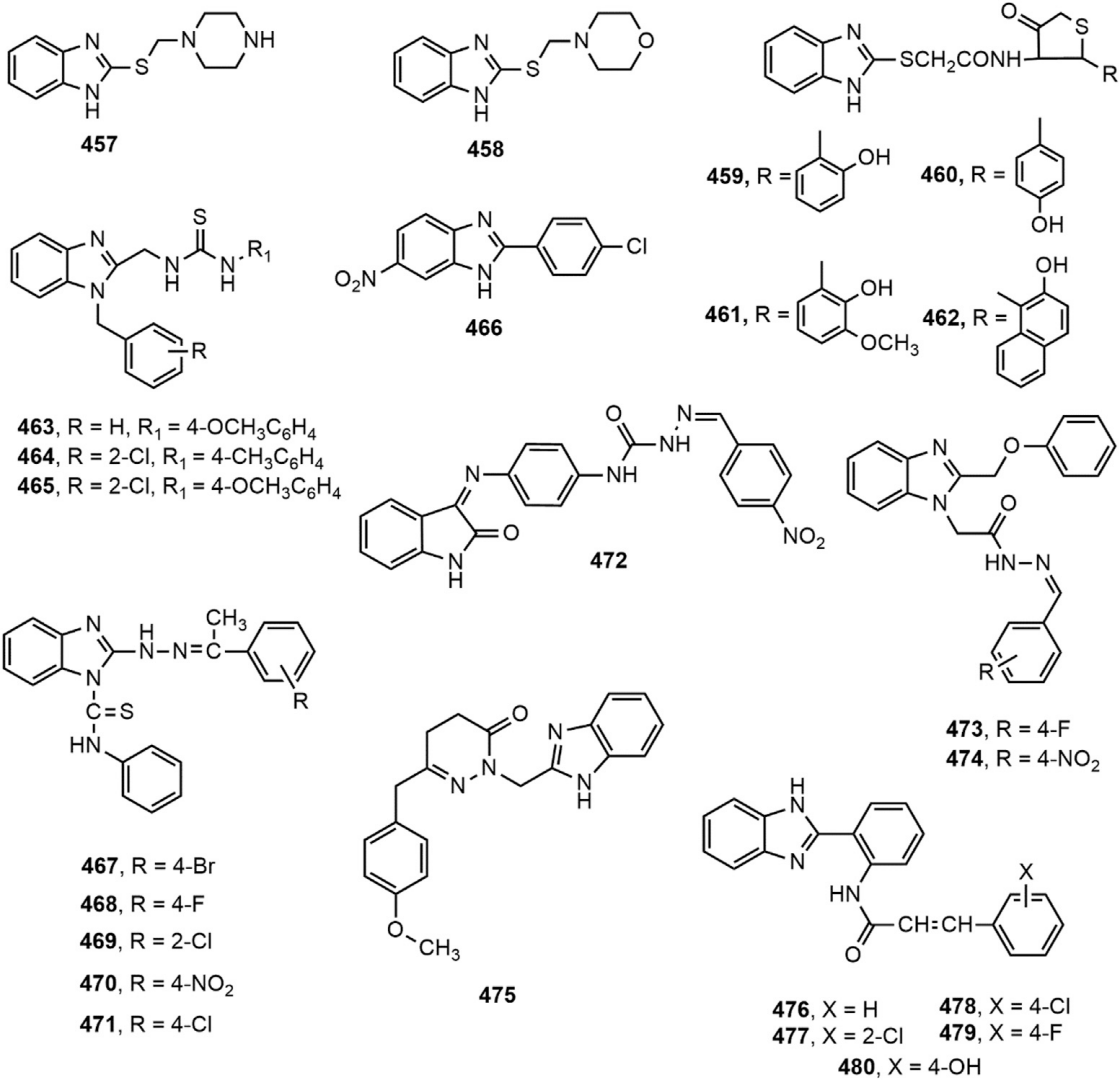
Benzimidazole derivatives with anticonvulsant activity.

Epilepsy is one of the most prevalent and serious neurological disorders, and recurrent seizures or convulsions are its characteristic syndrome. Around one-third of patients in the world show poor response to currently available antiepileptic drugs (Keri et al., 2015). In search of novel clinically effective anticonvulsant medications, benzimidazole nucleus has recently been explored by scientists with promising results. The benzimidazole derivatives with anticonvulsant property are shown in Figure 19.

A library of 2-mercaptobenzimidazole derivatives were evaluated for anticonvulsant activity using maximal electroshock seizure (MES) model. The synthesized compounds displayed anticonvulsant property at a dose of 20 mg/kg (i.p.) compared to standard phenytoin and compounds **457–458** appeared to be the most potent of all (Anandarajagopal et al., 2010). A series of 4-thiazolidinones and 1,3,4-oxadiazoles bearing 2-mercaptobenzimidazole moiety were assessed for *in vivo* anticonvulsant activity (Shingalapur et al., 2010). Compounds **459–462** emerged as the most promising anticonvulsants in MES model.

Siddiqui and co-workers described the synthesis of several 1-{(1-(2-substituted benzyl)-1*H*-benzo[d]imidazol-2-yl)methyl}-3-arylthioureas which have been mentioned earlier for their analgesic potential. The research group in a previous study reported about the anticonvulsant and cytotoxic effects of the same series of compounds. Compounds **463–465** were found to possess potent anticonvulsant property in comparison with standard drug phenytoin (Siddiqui and Alam, 2010). A series of nitro-benzimidazole derivatives were synthesized by Jain et al. and screened for anticonvulsant activity using MES and subcutaneous pentylenetetrazole (scPTZ) models. Compound **466** displayed the most promising result in inhibiting convulsion induced in mice by both methods (Jain et al., 2010). Bhrigu et al. synthesized a library of 2-[(1-substituted phenylethylidine) hydrazine]-*N*-phenyl-1*H*-benzo[d]imidazole-1-carbothioamides (**467–471**) from the reaction of 2-mercaptobenzimidazole with hydrazine hydrate, substituted acetophenones and phenylisothiocyanate. Compounds **467–471** were found to be active compounds in MES and scPTZ models, and devoid of neurotoxicity (Bhrigu et al., 2012).

A series of benzimidazole substituted semicarbazones were synthesized and tested for anticonvulsant activity using MES model. Compound **472** at a dose of 50 mg/kg (i.p.) appeared to be the most potent among the derivatives (Rajak, 2015). In another study, some oxadiazole bearing benzimidazoles and several derivatives of 2-[2-(phenoxymethyl)-1*H*-benzimidazol-1-yl]-N0-[(Z)-phenylmethylidene] acetohydrazide (**473–474**) were evaluated employing MES and scPTZ methods. Compounds **473–474** were found to be effective anticonvulsant agents (Shaharyar et al., 2016). A new series of hybrid benzimidazole containing pyridazinones were developed to assess anticonvulsant property. Compound **475** emerged as an effective and safe anticonvulsant in both MES and scPTZ models. The compound also exhibited notable increase in GABA level (1.7-fold) compared to control which was attributed to its good binding property with the GABA_A_ receptor (Partap et al., 2017). Recently, Sahoo et al. (Sahoo et al., 2019) synthesized several benzimidazole derivatives and disclosed that compounds **476–480** exhibited notable anticonvulsant potency (around 70–80%) in comparison to standard drug phenytoin.

### Benzimidazole as Neuro-protective Agent

**FIGURE 20:**
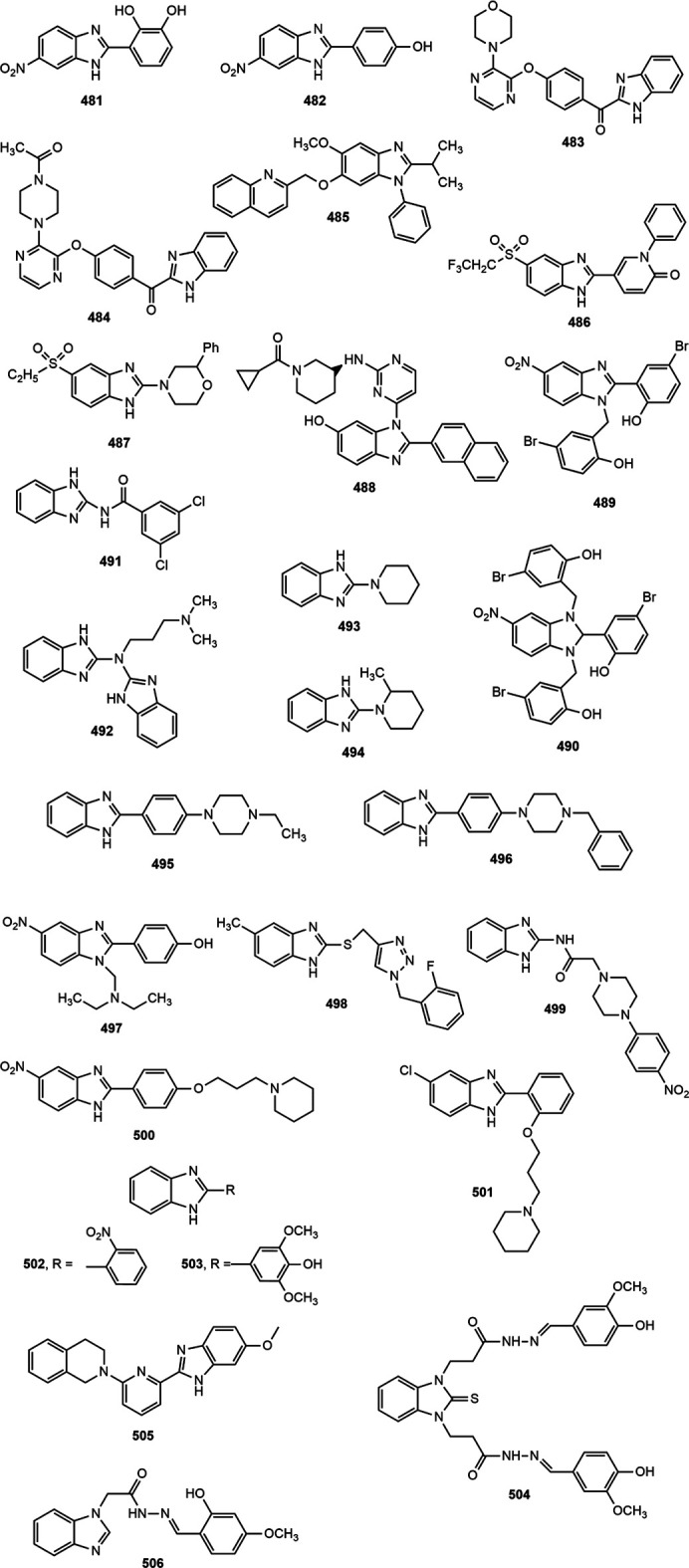
Benzimidazole derivatives as neuro-protective agents.

Neurological disorders comprise the disorders of central and peripheral nervous system. Alzheimer disease (AD), dementia, stroke, Parkinson’s disease, multiple sclerosis, brain tumor etc. are the most common diseases affecting a large number of people. AD is a chronic neurodegenerative disorder and the most common form of dementia manifested by loss of memory, language, cognitive functions, behavior and emotion. The factors which mostly contribute in the development of AD include the levels of acetylcholine (ACh) and deposits of neurotoxic amyloid-β-peptide (Aβ) (Akhtar et al., 2017). A number of substituted benzimidazole derivatives have been developed for the management and treatment of neurological diseases. The benzimidazole derivatives explored recently as neuro-protective agents are shown in Figure 20.

Khan et al. synthesized a series of 6-nitrobenzimidazole derivatives and screened their phosphodiesterase inhibitory property. Among them, compounds **481** (IC_50_ = 1.5 ± 0.043 µM) and **482** (IC_50_ = 2.4 ± 0.049 µM) were found to possess better activity than the standard EDTA (IC_50_ = 274 ± 0.007 µM). The presence of 2,3-dihydroxyphenyl (**481**) and 4-hydroxyphenyl (**482**) moiety might have contributed for their notable property (Khan et al., 2012). Hu et al. designed and synthesized a series of keto-benzimidazole derivatives (**483–484**) as potent and selective inhibitors of Phosphodiesterase 10A (PDE10A). Compound **484** showed 55% receptor occupancy (RO) of PDE10A at 30 mg/kg p.o. in rat brain. Further research led to the identification of compound **484**, with improvements in *in vivo* efficacy (57% RO in rats at 10 mg/kg p.o.), rat clearance and oral bioavailability (Hu et al., 2013). Hamaguchi et al. (Hamaguchi et al., 2013) prepared a series of benzimidazole derivatives as PDE10A inhibitors with reduced CYP1A2 inhibition among which the compound **485** appeared to be the most prominent.

Tamura and co-workers synthesized a series of benzimidazole derivatives for neuropeptide Y (NPY) receptor antagonistic activity which is an important pharmacological target for the treatment of different neurodegenerative disorders. Compound **486** appeared to be the most promising agent (Tamura et al., 2012a). In another study by the same research group (Tamura et al., 2012b), compound **487** was reported to show high NPY Y5 receptor binding affinity along with good absorption, distribution, metabolism and elimination (ADME) profile resulting in notable *in vivo* efficacy as NPY Y5 receptor antagonist.

Kim et al. evaluated a series of 1-heteroaryl-2-aryl-1*H*-benzimidazole derivatives as inhibitors of c-Jun N-terminal kinases (JNK3), a group of degenerative signal transducers and potential target of neurodegenerative diseases, e.g. Alzheimer’s and Parkinson’s diseases. Majority of the compounds exhibited high affinity (K_d_ = 10 µM–46 nM) to JNK3 when investigated through SPR, JNK3 kinase assay and cell-viability of human neuroblastoma cells. The most potent compound **488** showed notable cell protective effect (IC_50_ = 1.09 µM) against toxicity induced by anisomycin (Kim et al., 2013).

Human Presequence Protease (hPreP) is a mitochondrial metalloprotease capable of degrading amyloid-β peptide and increasing its proteolysis in human neuronal cells. Identification of potential agonists of hPreP is of great importance for Alzheimer’s drug design. Compounds **489–490** showed marked enhancement of hPreP-mediated proteolysis of Aβ, pF1β and fluorogenic-substrate V and thus presented great potential in the treatment of Alzheimer’s disease (Vangavaragu et al., 2014).

Among the therapeutic agents used for the treatment of traumatic brain injury (TBI), positive allosteric modulators (PAMs) of metabotropic glutamate receptor 5 (mGluR5) are important. He et al. (He et al., 2015) designed and synthesized a series of acyl-2-aminobenzimidazole derivatives based on the chemical structure of a well-known mGluR5 PAM called 3,3’-difluorobenzaldazine (DFB). The compounds were tested for binding affinity to transmembrane domain of mGluR5 using nitric oxide (NO) production assay, among which compound **491** (IC_50_ = 6.4 µM) was found to be around 20 times more potent than DFB (IC_50_ = 136 µM) (He et al., 2015).

Zhu et al. synthesized a series of 2-aminobenzimidazole derivatives (**492–494**) under microwave irradiation and assessed their acetylcholinesterase (AChE) and butyrylcholinesterase (BuChE) inhibitory activities. Compounds **492–494** displayed more than 25-fold more selectivity towards BuChE than AChE (Zhu et al., 2013). In another study, the compounds **(495–496)** showed selective inhibition on BChE and appeared to be the most potent BuChE inhibitors (IC_50_ = 5.18 and 5.22 µM, respectively) (Ozadali-Sari et al., 2017). Besides, compound **497** was found to be the most potent AChE inhibitor (IC_50_ = 0.93 ± 0.04 µM) with marked selectivity ratio (13.68) (Alpan et al., 2017). Similarly, compound **498** carrying a methyl group at 5-position of benzimidazole ring and 2-fluorobenzyl group connected to 1,2,3-triazole system was found to be the most potent inhibitor of AChE displaying 84% inhibition at 100 μM concentration (Faraji et al., 2017). The nitrophenyl piperazine substituted derivative **499** showed 57.25 and 77.92% inhibition of Rho-associated protein kinase II (ROCK II) enzyme at a concentration of 0.5 and 1 mM, respectively and exerted prominent IOP lowering effect (51.56%) compared to the standard fasudil (Abbhi et al., 2017).

Recently, compounds **500–501** were reported as promising AChE and BuChE inhibitors (IC_50_ = 0.14 and 0.22 μM, respectively), highly neuroprotective against hydrogen peroxide mediated toxicity, metal chelators, and free radical scavengers in the treatment of Alzheimer’s disease (Sarıkaya et al., 2018). Similarly, compounds **502–503** demonstrated duel effects as anti-Alzheimer agents by beta amyloid cleavage enzyme-1 (BACE1) inhibition and neuroprotection (Gurjar et al., 2020). In a 6-hydroxydopamine (6-OHDA)-induced oxidative stress *in vitro* model assay, compound **504** exerted neuroprotective action compared to melatonin, and reduced superoxide anion radical and hypochlorite level in a luminol-dependent chemiluminescent assay (Anastassova et al., 2020). In another study, compound **505** displayed significant anti-neuroinflammatory property (IC_50_ = 5.07 mM to prevent nitric oxide generation) and 65.7% hBACE1 inhibition. Besides, the compound (**505)** increased glutathione (GSH) level, reduced ROS production, and subsequently opened a door for further development to be established as a promising treatment option against Alzheimer’s disease (Fang et al., 2019). Finally, compound **506** exhibited promising neuroprotective role on SH-SY5Y cells by preserving the synaptosomal viability and minimizing GST level compared to the standards melatonin and rasagiline (Anastassova et al., 2021).

### Miscellaneous Activities

**FIGURE 21:**
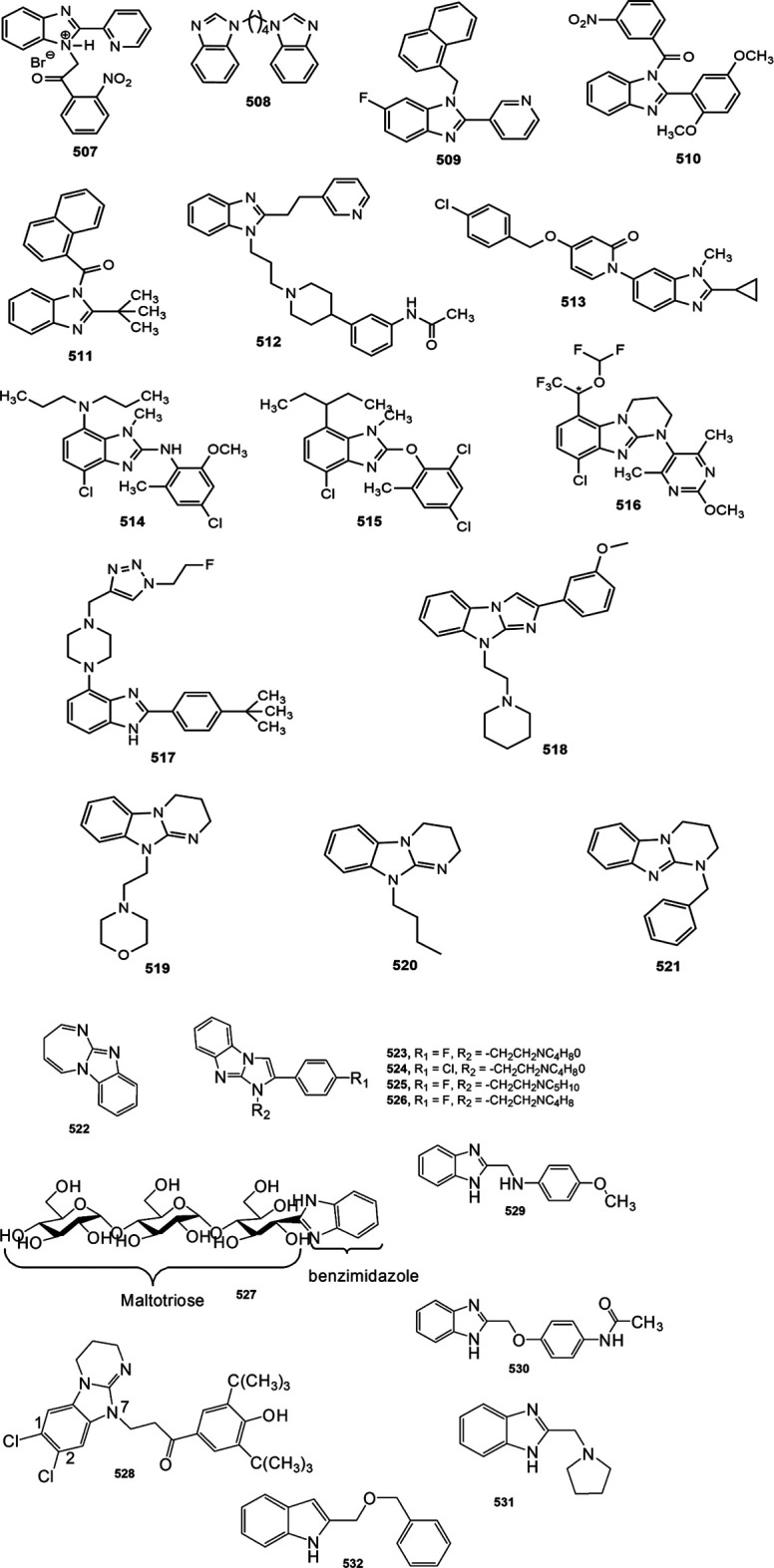
Benzimidazole derivatives with miscellaneous activities.

Several other classes of benzimidazole derivatives have been prepared by different scientists during the last few years. Some of these novel benzimidazole based compounds are shown in Figure 21.

Saify et al. (Saify et al., 2014) synthesized a series of 2-(2’-pyridyl) benzimidazole derivatives and evaluated their urease inhibitory property. Compound **507** displayed significant urease inhibition compared to the standard thiourea (IC_50_ = 19.22 ± 0.49 µM and 21.00 ± 0.01 µM, respectively) and twofold more activity than another standard acetohydroxamic acid (IC_50_ = 42.00 ± 1.26 µM). Several 1-alkylbenzimidazoles and 1,3-dialkyl benzimidazolium salts were evaluated for their tyrosinase inhibitory activity among which compound **508** emerged as the most potent derivative (IC_50_ = 0.31 mM) (Karatas et al., 2014).

A series of 2-pyridylbenzimidazole derivatives were designed and synthesized as Cannabinoid 1 (CB1) receptor agonists amongst which compound **509** appeared to be the most potent with a binding affinity value (K_iCB1_) of 0.53 nM (Mella-Raipán et al., 2013). The same research group synthesized a series of N-acyl-2,5-dimethoxyphenyl-1H-benzimidazole derivatives and assessed them for human CB1 receptor binding affinity using competitive binding assays (Espinosa-Bustos et al., 2015). Compound **510**, carrying 3-nitrophenyl group in the aroyl region of benzimidazole moiety was found to be the most promising agent (K_i_ = 1.2 nM). More recently, they synthesized a new series of benzimidazole derivatives which displayed more selectivity towards human Cannabinoid 2 (CB2) receptor than that towards CB1 receptor (Romero-Parra et al., 2016). Compound **511** demonstrated the best binding affinity (K_i_ = 0.08 µM), high selectivity index (K_i_CB1/K_i_CB2 >125) and low toxicity.

Lim et al. synthesized a series of 2-heteroaryl substituted benzimidazole derivatives bearing piperidinylphenyl acetamide group at 1-position and screened them for their effect on melanin-concentrating hormone (MCH), an attractive target for developing anti-obesity agents. Compound **512** displayed prominent MCH receptor 1(MCH-R1) binding affinity (IC_50_ = 1 nM), as well as low human ether-a-go-go-related gene (hERG) binding affinity thereby ensuring low risks of cardiovascular diseases, metabolic stability and preferable pharmacokinetic profile (Lim et al., 2013). Igawa et al. synthesized several 1-(1H-benzimidazol-6-yl) pyridin-2(1H)-one compounds among which compound **513** showed most prominent antagonistic property against MCH-R1 (Igawa et al., 2016).

Mochizuki and co-workers (Mochizuki et al., 2016) assessed several benzimidazole analogues for *in vivo* corticotropin releasing factor type 1 (CRF1) receptor antagonistic property. Compound **514** with an electron withdrawing cyano group at the 4-position of benzimidazole nucleus emerged as the most promising among the series. In continuation with their research to develop CRF1 receptor antagonists, compound **515** was reported as a potent CRF1 binding inhibitor (IC_50_ = 4.1 nM) and *in vitro* CRF1 antagonist (IC_50_ = 44 nM) (Mochizuki et al., 2017). More recently they designed a series of 1,2,3,4-tetrahydropyrimido[1,2-a]benzimidazole analogues as novel CRF1 receptor antagonists. Compound **516** displayed very prominent CRF1 receptor binding activity (IC_50_ = 58 nM) and oral bioavailability (F = 68% in rat model) indicating its potential in developing clinically effective CRF1 receptor antagonist in the future (Kojima et al., 2018).

A library of small molecules were tested for their property as human gonadotropin releasing hormone (GnRH) receptor antagonists. The derivative **517** (K_i_ = 13.8 nM) exhibited highest affinity towards human GnRH receptor and emerged as the most prominent GnRH receptor antagonist (Fjellaksel et al., 2017). Furthermore, a recent study reported several benzimidazole analogs **(518–521)** as intraocular pressure (IOP) reducer in the treatment of dexamethasone-induced ocular hypertension. Among these, compound **521** exerted the best anti-glaucoma action by the maximum IOP reduction of 22.32% from baseline following single drop administration (0.1%) (Marcus et al., 2019).

Maltsev et al. (Maltsev et al., 2020) evaluated a series of Diazepino[1,2-a]benzimidazole derivatives for their possible psychotropic properties. Compound **522** at a dose of 2.34 mg/kg showed prominent anxiolytic, antidepressant and anticonvulsant activities in both rat and mice models. Vasil’ev et al. (Vasil’ev et al., 2017) investigated a series of imidazo[1,2-a]benzimidazole derivatives using corazole-induced seizure model. The compounds **523–526** displayed notable anticonvulsive activity in comparison with standard valproic acid. The prominent activity is attributed to the presence of a 4-fluoro substituent in the 2-position and a dialkylaminoalkyl or cycloalkylaminoalkyl substituent in the 9-position of the fused ring.

Change et al. (Chang et al., 2017) investigated the antiplatelet activity of some novel saccharide-based benzimidazole derivatives. Compound **527** exhibited concentration-dependent inhibitory property against thrombin (0.01 U/ml) and collagen (1 µg/ml)-induced human platelet aggregation in an *in vitro* model. Its inhibitory effect might be attributed to the presence of 1-imidazolyl moiety at one end carrying a long chain of three sugar moieties. A series of benzimidazole derivatives bearing a sterically hindered phenolic group in their structures were evaluated for *in vitro* antiplatelet activity using the Adenosine diphosphate (ADP)-induced platelet aggregation model of rabbit’s plasma (Spasov et al., 2020). Compound **528** showed notable antiplatelet activity by exceeding the standard acetylsalicylic acid by 21.8 times. In the *in vivo* study of inhibition of intravascular platelet aggregation, the same compound displayed 1.5 times superior activity than acetylsalicylic acid and slightly inferior activity than another standard clopidogrel.

Idris and his co-workers (Idris et al., 2019) reported that benzimidazole derivatives **529** and **530** decreased repeated morphine administration-induced thermal hyperalgesia and tactile allodynia as well as the expression of spinal Tumor Necrosis Factor-alpha (TNF-α) in Balb-c mice. The compounds displayed PPARγ agonist activity, indicating their potential in the management of morphine-induced paradoxical pain. Akhtar et al. (Akhtar et al., 2021) evaluated the role of benzimidazole derivative **531** in managing nalbuphine-induced tolerance in cisplatin-induced neuropathic pain in mice. Cisplatin, a platinum-based anticancer medication, is often coupled with Nalbuphine, an opioid analgesic used for treating acute and chronic pain. The compound **531** attenuated the tolerance to the analgesic activity of nalbuphine developed due to its long-term use, as well as TNF-α expression in the spinal cord of mice.

Moreover, Raka et al. (Raka et al., 2021) reported a series of substituted benzimidazole derivatives and found a notable inhibitory property of compound **532** against the acetylcholinesterase enzyme with an IC_50_ value of 29.64 μg/ml compared to the standard drug donepezil (IC_50_ = 9.54 μg/ml).

## Expert Opinion

Heterocyclic compounds possess versatile applications, such as antibacterial, antiviral, antitubercular, anticancer, anti-inflammatory, analgesics, herbicidal, fungicidal, insecticidal, antidiabetic, antihypertensive, and so on (Pathan et al., 2020). Some of these heterocyclic compounds, having several photochromic, solvatochromic, and biochemical luminescence characteristics, are widely distributed in natural sources as in plant alkaloids, hemoglobin, anthocyanins, flavones, etc., and play a vital role as drugs, vitamins, chlorophyll pigment, dyes, amino acids, and enzymes in human life (Pathan et al., 2020).

Among the heterocyclic compounds, benzimidazole derivatives have been playing the most pulsing and eye-catching pioneer role in synthetic pharmaceutical and agrochemical sectors for the last couple of decades (Pardeshi et al., 2021). As having the similarity of the benzimidazole nucleus with many naturally occurring nucleotides and its presence in several natural compounds, the benzimidazole derivatives can easily interact with various biomacromolecules or target proteins. Thus, the compounds derived from the benzimidazole ring system exert broad-spectrum efficacy against various human disorders like cancer, hypertension, diabetes, bacterial or viral infection, inflammation, gastritis, neurodegenerative disorders, and so on (Wang et al., 2015).

Moreover, the first effective therapy developed through benzimidazole moiety against the Ebola virus was an innovative breakthrough (De Clercq, 2019). Categorically, several approved drugs derived from benzimidazole are currently available in the market and they can be grouped as *1*) proton pump inhibitors (PPIs) exemplified by omeprazole, esomeprazole, lansoprazole, pantoprazole, etc., *2*) anthelmintic drugs demonstrated by mebendazole, albendazole, oxibendazole, etc., *3*) non-sedative H_1_-receptor blockers or antihistamine presented by astemizole, norastemizole, emedastine, mizolastine, etc., *4*) angiotensin II receptor blocker illustrated by telmisartan, candesartan, azilsartan, and *5*) calcium sensitized cardiac agents instanced by isomazole (Tahlan et al., 2019).

As stated in the comprehensive review, there are diverse classes of benzimidazole derivatives synthesized by several groups, which showed prominent bioactivities even sometimes better than the existing drugs. Although several structure-activity relationship studies were accomplished by several groups in a particular area of bioactivities, it is challenging to draw any conclusion on the structural features needed for a specific activity. In many cases, further studies on specific benzimidazole derivatives leading to the development of potential drug candidates were not continued. The synthesis of a new library of benzimidazole structures is expanding day by day, with a unique spectrum of bioactivity being reported regularly. Despite having extensive biological and diagnostic applications with the lustrous futuristic potentiality of benzimidazole scaffolds, barriers and challenges are still viable in the clinical development process. Drug resistance is the major drawback of the antiviral, antifungal, antibacterial, antimalarial, anti-cancer, anti-hepatitis, and anti-HIV agents (Wang et al., 2015). Another notable concern is that several benzimidazole derivatives such as PPIs are showing *in vitro* and *in vivo* drug-drug interactions with several classes of therapeutics such as anti-cancer (Bezabeh et al., 2012) and antidiabetics (Hossain et al., 2020; Hossain et al., 2021).

Benzimidazole derivatives containing pyrrolidine side-chain exhibited a beacon of hope in malignancy treatment where several available drugs (for example, sorafenib) have become resistant due to long-term exposure against several cancers, such as hepatocellular carcinoma (Suk et al., 2019). It is a crucial fact that benzimidazole derivatives have been synthesized and screened against many disease conditions; among them, very few enter into clinical trials. For example, selumetinib (NCT01933932) and galeterone (NCT04098081) are two promising agents undergoing several clinical trials to be established as effective cancer agents. Besides, several ongoing clinical trials (For instance, NCT01611974) of various drugs (maribavir) bearing benzimidazole moiety showed promising effects against resistant cytomegalovirus (Maertens et al., 2019; Papanicolaou et al., 2019). Dexlansoprazole completed the phase IV trial (NCT01093755) and showed extensive effect against esophageal inflammation (Sharma et al., 2020). However, these reported trials are still very negligible compared to the preclinical status of the ample amount of benzimidazole drug candidates.

It is crystal clear that an exciting number of benzimidazole pharmacophore derivatives have become potential drug candidates to treat several disease conditions in ongoing clinical trials. However, this promising moiety is copious to be further investigated for designing a number of different target molecules, which might reveal some encouraging outcomes in medicinal research. It can easily be assumed at the current stage that several new strategies might be designed and developed for multi-target agents with the highest affinity, specificity, and enhanced bioactivities. In the future, the benzimidazole scaffolds might be fused with another heterocyclic ring system to be explored several novel chemically stable and potent pharmacological agents against several rare or critical diseases or with better pharmacokinetic and pharmacodynamic profiles. This might be an outrageous and worldwide revolutionary achievement in the next century. Toxicity, drug resistance, and poor bioavailability are the significant concerns during drug development or the underlying reasons behind the compounds be untouched in the human trial. Structural modifications of the existing synthesized molecules might be an excellent technique to be brought about improved or novel activities with a better safety profile. Besides, the prodrug strategy can also be applied to ameliorate the bioavailability of the present derivatives. Furthermore, combinations of the benzimidazole derivatives with other existing drugs might be explored for finding synergistic effects or exciting pharmacological activities against multidrug-resistant microorganisms.

Moreover, several benzimidazole-containing clinically used medications, e.g., omeprazole, lansoprazole, pantoprazole, and rabeprazole, are actually in the form of prodrugs for which the onset of action is slower. Thus, there is an increased demand for producing the active form of drugs directly from benzimidazoles, and for this, researchers are paying attention to novel benzimidazole-derived therapeutics with lesser toxicity and better pharmacodynamic profile. In this case, PPIs containing benzimidazole ring might be modified, or the benzimidazole pharmacophore might be replaced by imidazole ring with the improved biological profile. The non-approved benzimidazole scaffolds for cancer therapy having eminent pharmacokinetics and safety profile might be repurposed to treat several life-threatening cancers with a lack of safe and effective treatment. Some anthelmintic drugs containing benzimidazole nuclei, such as flubendazole, albendazole, and mebendazole, could be considered to apply against various types of cancer in future investigations. Besides, stereochemistry could be regarded as to trace and explore more potent isomers of the existing approved benzimidazole derivatives because one enantiomer may exert distinct biochemical property than the other enantiomer.

## Conclusion

Benzimidazole, an essential nitrogen-containing heterocyclic moiety, can be found in various therapeutically used compounds playing a vital role in treating many diseases. Many efforts have been given so far on the development of target-based benzimidazole derivatives, and the interest to produce new therapeutically active agents to treat different diseased conditions has grown noticeably during the last few years. However, this field has several challenges, especially to bring the numerous synthesized compounds into the clinical trial, which showed valuable pharmacological properties in different studies, and later to ensure their availability in the market and clinical practice. The present review has focused on the current status of benzimidazole moiety, emphasizing the SAR of different benzimidazole-based compounds explored by scientists around the world. So far this is the most inclusive and informative account about biological and therapeutic potential of benzimidazole derivatives. With a compilation of information from more than 250 latest pieces of literature, we aimed to aid the researchers, medicinal chemists, and drug designers with valuable and comprehensive knowledge and provide them the rationale to develop target-oriented and clinically useful benzimidazole-based molecules.
